# Supplementary material for: Does supporting self-help groups of people with mental conditions for longer duration lead to more effective groups? A qualitative evaluation in Ghana
Source: PLOS Ment Health. 2025 Jan 30;2(1):e0000249. doi: 10.1371/journal.pmen.0000249 (PMC12798605; doi:10.1371/journal.pmen.0000249)
Supplement: S1 Data — (PDF) [file pmen.0000249.s001.pdf]

ANONYMISED TRANSCRIPTS

Ice Breaker

My concern is about the people on the street with mental health condition. I was wishing if all our health care and medicines were on health insurance in that case if anybody at all see somebody on the street with mental health issue the person can just bring the sick person to the hospital knowing they're not going to pay for any services

Operational structures within the community

Q: How does your SHG operate within your community?

Response: I can say that our meeting are **once in every month** but the participation is not all that good because in my group like this not everyone in the……. district come to the meetings those who are a bit far away like Spintex find it difficult to come because of money issues, it is not a walking distance whereby they can walk to meetings and so they are asking for financial support to help them come to meeting regularly.

Q: Describe your typical day as someone with mental Health condition or disability to me

Mostly I am on medication. And since some people are not stable, it is their care givers that is taking care of them and we those that are working sometimes you don't feel good although you are on medicine it is not every day that the medicine works. It can't be like you were before so sometimes you won't feel like working but because you are under somebody you can't give excuses every day and so you're forced to come every day. So even though you are on medication you still have to go to work every day just like any other person.

I can say when people like basic needs and MEHSOG visit us because occasionally MEHSOG gives us outreaches like they bring doctors, psychiatrist pre-medications and those stuff they're happy about that because some people cannot afford the medicines and so when they hear free medication they are happy and at times basic needs gives seed money for those who are not working some even get fridges and popcorn machines to go and work and I think these are the things they like in the group

I: What are some of the unpleasant experiences you have?

Response: Some of the unpleasant things they experience I think mostly is stigma and people still believe that the condition is spiritual instead of seeking help at the hospital they will be going to prayer camps thinking that they will be healed. It is good to pray but in addition come to the hospital but some people don't come to the hospital until the situation becomes worse before they come to the hospital. I know of a member who loves to spend her money on food and not on medicines and so that is why I am stretching that if all our medicines are on the NHIS we will be stable and work and live a dignified life

Current Financial Operations

Q: Please tell me about the operations of SHGs

Response: Before the covid-19 was but now we do not receive apart support from the outreaches that they do and give free medicines and before the covid we were not receiving support frequently I can only remember one occasion when they came to give us the seed money and refrigerators and some stuffs but if they organize a program for us they give us T & T

The last time we had support was **from Basic needs and MEHSOG** and as **MEHSOG** it was early this year we received support from them they brought medicines for us.

Collaborations with Government Institutions

We've not been collaborating until the social welfare accepted us to be part of the district common fund and most of us have applied but nothing has happen, they keep telling us there is no money and the people who were there before we joined have not been settled and so they are not even thinking about us this year unless maybe next year when the funds are .Our last collaboration was with social welfare together with Basic needs at their premises as well as NHIS

Some of the action points we raised were about putting our medicines on health insurance and making us part of the district assembly common fund. Those were the main things we discussed, and we did follow up, but they tell us there is no money

Community Involvement

The community is not involved, and we have not been consulted by any benevolent person in the community to support

We have done several health campaigns with basic needs, and we use to do them quarterly but currently they say there is no money and so they do it twice a year instead of the four times

Some of the benefits of the health campaign we organized have created awareness to people who think their condition is spiritual to now believe that mental health condition can be manage through medical treatment and now they have become well.

I remember one sister who has relocated to the village when she relapsed a man slept with her and she became pregnant and the man lives in the vicinity but doesn't want to own up because the girl has mental health issue and it now the problem of the girl and her family and she was living in someone's room and her time was due but since her sister could no longer afford the rent bill she was compelled to leave to the village and now she is unable to get medication because there is no job over there in the village

We also face stigma problems as well when someone realizes you have mental health issue they don't want to employ you because of the relapse they get at the work place

Facilitators and Barriers

Support and unity. Financial difficulty works against the group because there are so many things we cannot do.

Recommendation

The government must initiate a policy that will give people with mental health issue the freedom to work, and the government should also ensure that our medicines are also included in the health insurance and build our capacity
So far, we have not received any skill training from anywhere. The good news is that we are competent enough to know our medications and when to take them

We urge everybody to do something to support themselves so that people will respect them rather than begging people for help and losing their dignity through that

Interview
Ice Breaker
One thing dear to my heart i want the government to do for us is to provide free medicines to people with mental health conditions in the country

Operational Structure Within the Community
Thank you. We meet every first Sunday in the month from 3pm to 4pm. We do the meeting briefly because some of the members become weak and sleep a lot when they take their medicines and some also have their personal things they do

Sometimes it is difficult to converge on them at the meeting place where I have to move from house to house to remind them about our meetings

some of the pleasant experiences we get is when people involve us in their activities, and it has help us to now socialize with people in the community more unlike before when we were introverts and we can't come around or associate with people

some of the unpleasant experiences we get as people with mental health issue is the stigma we go through in the community like people saying ill comment about such as calling us mad man or mad woman

if people in the community should approach us and ask us what is wrong with us we will open up and tell them about our condition but when they see us acting abnormal or relapsing they make ill comments about us we become embarrassed and as a result we can't come close to them

current Financial Operation
we don't receive support frequently, occasionally we receive support from Basic needs or MEHSOG

Sometimes they invite us to their office for brief discussions and during the meeting they serve breakfast and lunch as well as money for transport and upkeep

The last time we received support was Basic needs and MEHSOG. Three months ago they organized a free health care at the community hospital and free medicines as well and since then we have not receive any support again

Collaborations with Government Institutions

Occasionally the government institutions will invite the leaders to their premises for a discussion and we also use such opportunities to tell them about our needs and they will promise to help us, but we don't see any positive outcome

SELF HELP GROUP

Ice Breaker

Over here in this community most of the people are hawkers. However majority of the members in my group are aged people who cannot hawk on the street like the youth do and so we are pleading with the government to support our aged people with capital and electronics like refrigerators to start house businesses like selling of sachet waters, sweets and ice cream to cater for their selves

Operational structures within the community

Q: How does your SHG operate within your community?

Response: We used to meet **every Thursday** but it wasn't favoring the majority at a point and so we change the meeting days to last Thursday of every month and the members participate a lot during meetings

As for the living condition i can't it is ok but in all we thank God for keeping us alive because some of the members don't like to take their drugs and so their care givers must always influence them to take their medicines. Because medicine increases their appetite for food they don't like to take their medicines because they don't have enough money to buy food

Some of the unpleasant things they experience is when they don't receive free medicines and donations from organizations and also the stigma we go through as people with mental health conditions such as people calling us mad people and we use to get worried before when people address us like that but we grow to ignore such comments from people in the community

Current Financial Position

We don't receive support frequently; support comes when we meet with NGOs. Sometimes we get funds from a benevolent lady who pays some of our members hospital bills when they go health facilities and also their medications

Initially we used to contribute little money like 50 pesewas and 1 cedi and give it to one person to start business with and later pay it back to us and we will give the payment to another person to also trade it but currently we have stop because of the poor economic condition in the country the people are unable to pay back the money within the given time

The last time we receive support was early this year from an individual benevolent person who gave two of our members funds to start trade and **Basic needs** also gave us funds and also an individual woman called Ann gave us money during the Ramadan festival.

Collaboration with Government Institutions

We met with the Social Welfare Department I think early this year and NHIS also met with us some time ago I can't remember they registered our members into the NHIS and also renewed some of us our cards.

Community Involvement

We don't organize health campaigns in the community but what we do is that when people ask us about our condition then we try to educate the person about our condition. I personally advise families who have relatives who are suffering from mental health condition to send them to the health centers for treatment

Initially we used to face challenges accessing health care such as purchasing medicines at the health facilities were difficult
We received support from Basic needs and sanitary pads from family planning Association

Challenges

Some challenges we face is the membership attendance to meeting i have to be influencing them always to come to meetings. We are mostly stigmatized. People do not want us to be where they are because of our illness.

Facilitators and Barriers

Our supporters such as Basic Needs and MESOGH are our hope. We are stigmatized and not respected. Family and friends also neglect us.

Recommendation

Some measures to protect us are that the government must create a policy that will prosecute people who harm or violate us.
The government must also provide all our medicines for us free
The government must also establish small businesses for us so that we live a dignified life people will respect us when they see us working

IDI TRANSCRIPTION FOR THE SHG

Ice breaker

Our problem in this community is that we have neighbors who have mental health conditions and are being chained and detained at shrines and mission houses and are abandoned by families and so i formed an alliance with one of my locals called Theophilus popularly known as Bro T.T who work at the medical department under the Ghana fire service to gather people with mental health conditions in the community and solicit for support from NGOs and inform government agencies of our intentions and how they can provide support to the people with mental health conditions . fortunate for us we got an NGO called Basic needs and mental health society of Ghana (MEHSOG) who agreed to support us, we had our first meeting with these agencies at the Ussher polyclinic which is currently Ussher hospital and during the meeting we were divided into community self-help groups due matters which were raised and major among them was distance which resulted into disintegrating us into groups. However, we the leaders of the various groups merged and formed the ……District Assembly for people with mental health conditions

We have been receiving help from Basic needs but their support are not adequate and so we are pleading with the government and other NGOs to come to our aid and support us with medications to curb our condition so that we can work to support ourselves because the medicines the government donate to us are not sufficient and as a result we are forced to buy the rest with our own money and we find it difficult to buy them because we can't work

Operational structures within the community

Q: How does your SHG operate within your community?

Response: Thank you, as I said earlier, the various groups meet **once every month.** Initially the Gbese self-help group use to meet at a place close to the high street but someone is now trading at the place and so we informed the assembly man popularly known as Alhaji Boye Issah Quartey and he gave his office to us for our meeting place and accepted to pay all utilities bills that will come up. **We meet every Sunday of the month**

The Asere self-help group also **meet in the family house of one member by the name Florence dodou** close to a printing press across the highway. They meet last Wednesdays in every month whiles the **Atukpai self-help group meet one Sunday** after church service in every month

……. also meets at the palace court after consultation with the chief through the help of a member who is a niece to the chief and they meet Tuesday once in every month. Zongo lane self-help group use to meet in a noble man house but since the demise of the man the family stopped them from meeting in the house and currently, they meet at the open field in Zongo lane. Basic needs donated chairs to us many years ago but many of the chairs are spoilt and so any time we meet we have to

rent chairs to sit on. Before the covid-19 our members were very regular at meetings, they come in their numbers and they participate during discussions, however the attendance has now reduced. Some of our members use to work but because their condition triggers at any point in time people have stop employing them and this is as a result of their inability to afford their medication

Some of the unpleasant experiences we have had as people with mental health condition is the stigma we go through like some people in the community address us mad people and this is deteriorating to us because we become sad whenever we being teased and address in that manner
Some of the pleasant experiences we have had are when our relatives accept us and protect us from people who try to harm us. Another thing is the donation and livelihood support we receive from NGOs like Basic needs and MEHSOG and this makes us happy.

Current Financial Operations

Before covid-19 we received financial support from Basic needs and MEHSOG every quarter in a year and noble people in the community. We met with the social welfare department and petitioned them to support us financially but unfortunate they supported only four members and since then we haven't received any support from them. The last time we received funds was 2019

Collaboration with Government Agencies

We met with social welfare department, sub metro and NHIS however our meetings were not frequent. When we meet with them and present to them our proposals they will tell us that they will work on it and give us feedback but all to no avail despite the follow up we do and the last time we met with a government agency was early this year with the sub metro at their office at …… and I remember we pleaded with them to advocate for us ensuring that the NHIS cover our entire medications

Community Involvement

Like i said because of the stigmatization we were facing in the community we organize a community symposium last November at …… palace to create an awareness in the community and this has reduced the stigmatization in the community. People have also come to the awareness of how their relatives with mental health can be stabiles through medical treatment and eradicate the perception that such conditions are caused by spiritual forces

I recommend that members of various self-help groups should make every effort to not respond to people who insult them but rather they should always look neat and work to support themselves as well as contribute little tokens to support any member when the need arises.

Facilitators and Barriers

The facilitators of SHG activities will be based on constant support. We are often stigmatized and isolated. Even sometimes family and friends neglect us, and they think our health condition is contagious

Recommendation

There should be a policy to give people with mental health issues the freedom to work. Mental Health medicines are also included in health insurance. Skill training will also help us.

Our members must do something to support themselves and not to be begging.

FOCUS GROUP DEMOGRAPHIC DATA

Interviewers: Good afternoon to you all. My name is ….. I came with my colleagues who are, my name is ……. We have come and take this information from you and he has also sent us to stand on his behalf to take this information. This afternoon we have a few questions for you. It will help us know what goes on in this group. When we start this group questioning, we'll give you numbers. When you are to respond to a question, mention your number and respond to the question. When I ask a question, any of you can raise your hand, mention your number, and give your response. I will start from my right side, my grandfather you would be number 1, you would be number 2, you would be number 3, you would be number 4, then number 5 and number 6. When you get up to answer a question don't mention your name, mention your number. Our first question is.

Interviewers: How do your SHGs operate within your community (ies)? · **Probe** for times/days of operation, membership, convening of meetings, frequency, participation during meetings, how resources are sourced, etc.

Respondent 1: I'm from the …….community and I'm part of it. The meetings we have are always scheduled every first Friday of every month.

Interviewer: Any other opinions?

Respondent 2: We are ….group and we are part of the group because of our health, and we are there because they always help us with drugs and the time, we will take their drugs and get well. Today we are working.

Interviewer: We would like you to tell us the time you have your meetings or days and when you sit for the meeting, what you always talk about, when you sit for the meeting what's your total number so if anyone can answer you can add your answer. Yes, my mother, you can answer. OK if you don't have the answer when they talk keep quiet.

Respondent 3: In this group, our meetings are always on the first Friday of every month.

Interviewer: So, when you sit for the meeting what is always your number?

Respondent 3: Our number is 20.

Interviewer: We are moving forward.

Interviewer: Describe your typical day as someone with a mental health condition or disability too. When you wake up how does your day look like? What time do you go to work, what time do you come from work and when you go to work what do you do?

Respondent 3: We are farmers. We don't go to the farm in the afternoon we go in the morning by the time the sun gets hot we will be back home because when the sun is scorchy it can trigger our sickness.

Interviewer: Good, any other addiction?

Respondent 4: As a person living with a mental health disability when I can wake up and I'm not feeling very well I have to sit down till I'm calm before I can get up. When I get up, I go home straight I will not let the sun scorch me.

Interviewer: Any other additions?

Respondent 5: Because I'm not well when I go to the farm and I'm farming my hand would be shaking, when that happened, I must sit down slowly and wait till I'm OK sometimes I have a child with me so when I'm struck with the sickness, she ties me down till I'm OK. immediately after I recovered, I would tell her Let's go home and we will go home.

Interviewer: What are some of the unpleasant experiences you have had as someone with a mental health condition or disability?

Respondent 3: My sickness doesn't like a lot of noise so when I see people gathered around and they are making a lot of noise if I go there, it will start

Interviewer: Any other.

Respondent 1: The disheartening thing about this sickness is that you can wake up and the sickness will start. when I'm not able to get money to buy drugs or when I lack money to get drugs it causes me a lot of distress because the sickness will start and that is the unpleasant thing about living with this condition. Most of the time it's caused by poverty.

Respondent 2: With my sickness sometimes fluid comes from my nose when we are gathered like this the fluid will choke me but I can't flush it out because of a lot of people I have to walk out to do it and I can't also swallow it back to my stomach I have to grow it out additionally when there is crowd somebody can just walk past you and you in here they are perfumed into your stomach it can trigger a sickness also, in my workplace when the sun is scorching I have to sit at a place of shelter or when I'm working with people and I realize this is going to trigger my sickness I will disassociate myself because if I force myself to work till the end of the work the sickness will be visible for everybody to see

Interviewer: Good, we are grateful to you all for opening. We are moving forward.

Interviewer: What are some of the pleasant experiences you have had as someone with a mental health condition or disability?

Respondent 2: What makes me happy is when I wake up in the morning and is still cold, I feel so good (laughing) but when the sun rises and you say Let me get up and do something small to get money than your body will be weak and it gives the sickness more power

Respondent 3: Because we are farmers we must get up and go to the farm early because when the sun rises and becomes scorching it triggers the sickness.

Respondent 1: About this sickness what makes me happy is that in the morning I have to check myself to see if I'm fit, if I'm done checking myself by 6:00 AM to 8:00 AM I will know my health status and know whether I can start work but if I realize I'm not in good shape I will leave the work

to God and pray to Him (God) because He takes care of His children, I will not stress myself and add more troubles to my troubles. That's how I observe my day when day breaks.

Respondent 6: My sickness starts when I'm sleeping or I'm about to sleep. It can wake me up from sleep and sometimes when I'm up I'll be talking to myself, When that happens and I am able to sleep back I will be OK Additionally Thursday, Friday are bad days for me.

Interviewer: I'm happy with the way you are answering the questions. We will move forward.

Interviewer: Please tell me about the operation of SHGs

· Probe sources of funds, funding stakeholders, frequency of receiving support, last time support of received and by whom.

Respondent 2: Where I get my money to do my work is in our group because they have taught us that we should work small, small. so that we can get something small to eat, when I go to get the drugs every day, I take one in the evening when that drug is finished and I'm not able to get it the sickness comes back.

Interviewer: Please, if anybody answers the question very well you can answer. It seems like we are deviating.

Respondent 1: What gives the groups money is when we agree in unity with those who are coming to support us. The support is not always much but we have faith and when you have faith and you are in this group, we can contribute one cedi each, Some time ago we instituted that but it didn't work. The contribution will help sustain us when the people supporting us are not able to support us again.

Interviewer: OK I'm not cutting you short, but do you get support from NGOs?

Respondent 1: OK we get support from Basic Needs.

Interviewer: Which period did they support you?

Respondent 1: The last time they supported us was four years ago before COVID-19 came and broke it. Sometimes they used to send someone from Accra to come and be at our meetings, but COVID-19 broke it and that was four years ago.

Interviewer: OK we are grateful

Respondent 3: Before we got any support, we used to contribute 1 cedi each. so that it can help when the support is delayed but now, we can't afford the 1 cedi again.

Interviewer: Good, let's continue

Interviewer: Now I want us to talk about our Collaborations with government institutions (district/municipal assembly, social welfare department, and national health insurance scheme)

Please tell me about how you collaborate with governmental stakeholders in mental health care. Do you frequently meet with the district assembly, social welfare department, and health insurance?

Respondent 1: Yes, we collaborate with district assembly, social welfare, and National Health insurance because there are times our health insurance will expire, and health insurance would let us gather all the cards and they will help renew it for us at no cost and this has happened twice and I'm a witness. Also, with social welfare, I witnessed one person in this group who was helped.

Interviewer: Which period did social welfare help him?

Respondent 1: that was four years ago, how that person got help from social welfare was when we heard there was a new common fund, and we heard people with disabilities have received help. some received grinding meal machines and got money to put up a structure for them, even to the point that some people went and attacked social welfare to help them get a tractor, They are a group at ….. and social welfare refused to adhere to their cry. We had a general meeting at ….. and that was where we had this information. One person among us got that support from the district assembly and that was when they were giving freezers, sewing machines, and cattle to people living with disabilities. Only one person among us got a fridge from the district assembly and by then our number was around 25. It was during this meeting we heard that people with mental health conditions also received support from the district assembly and there was tension. When we heard that we planned that we would go and demonstrate at the district assembly that coming Monday, I knew the information would be given to the director. So, when it was Monday, we picked our leaders to meet up with the director and ask him. It was during this meeting that one of us in this group received something from social welfare and not the district assembly. Also, it was once one of our members visited the district assembly and met them sharing freezers and he also got some by replacing someone who wasn't there, later Some of the group members came to ask me about it mostly nothing happens in this group without my knowledge so when they came to ask me about the fridge I told them I have no idea and when I later investigated I realized that he was also lucky, he went and met it at the district assembly so I explained to them that he was lucky so we should let it go. Social welfare didn’t relax there, they looked for the person whose name was supposed to receive the fridge and give him one and resolve the matter. These are the two things I've witnessed from the social welfare department.

Interviewer: Tell me the last time such collaboration was held, where, and who participated

Respondent 1: When you are talking about groups that have come to train us, basic needs have come to train us before. They came and taught us ways to get hand skills so that we would not be dependent all the time but they don't always come and pick some individuals out to learn soap making or making pomade, they don't always come and select some individuals from the group but they always support some of us with sewing machines and that is part of the skill training and you can’t ignore it. Basic Needs has done these things for us.

Interviewer: Good, let's continue to the next question

Interviewer: What type of training do you think this group needs so that it will help sustain its members or people living with mental health conditions?

Respondent 1: I will answer this question too.

Interviewer: What type of training do you think this group needs so that it will help sustain its members or people living with mental health conditions? So that it can give you money so that anytime you need money to buy something you can get your own money

Respondent 3: In this group if we get Support from a different source, we would have looked for support for ourselves.

Interviewer: What type of skills do you want?

Respondent 1: The skills that we need are. When we get support with money, we can look for the type of skills we want

Interviewer: clarify the question. What type of skills do you want from basic needs?

Respondent 1: There are different approaches to getting skills. When you support someone with money that's the skill you have given the person because when you bring the money you will not say we should take it and you just go you let us know what we are going to use the money for and it is acquiring new skill you can also let us know that when we are spending our money let's not forget about the bank because sometimes it's in the bank that will save our money

Interviewer: I'm not limiting you but this is how we want you to answer the question We would like you to clarify what exactly you want basic needs to train you on for example should basic needs train you on hairdressing or carpentry or weaving? that's what we need because if you are a hairdresser you can go and get money from that place so tell me what type of skills do you want for this group?

Respondent 1: This thing has worried us for a while now because it is, tailoring and carpentry. Some of our members are already in that business and they don't make any money, For example, imagine we all having tailors in our house who will give their clothes to others to sew so and Besides in this community when we have sewing machines they don't want to give us their clothes to sew because they believe we are mentally not sound some will not even give you money because they believe you are mentally not sound you don't know how to count money so it's not our will that we don't want the tailoring training but if we collect it, we can't use it to make money so it will not serve its purpose.

Interviewer: We have taken notice of your answer, any other addition?

Respondent 2: we need support. Support towards clothes weaving I want that type of skill additionally I'm a bicycle repair That's where I get something small from but when the sun gets scorching, I'm not able to work so usually I work under shelter.

Respondent 5: If the support is available for us, I don't stand in the sun for a long because when I’m hungry it triggers the sickness so I would say if I could get some of the lady's cooking stuff I could be doing that business while I'm inside the house so that anybody who needs to buy something can just walk in and buy. So, if I get money I will do business because if I step outside the sun it triggers my sickness.

Interviewer: Good I like the way you are answering the questions now to our next question

Interviewer: How do you involve the community in the operations of SHGs activities?

Probe for who in the community is engaged, how are they consulted/involved, when are they engaged, what roles the community members play, etc.

Respondent 1: With this question we have those we add to our lives just to promote unity some time ago they gave us a garden and they told us that when people who are not part of us want to help us we should allow them to help us so we didn't deny them we announced for them to come and help us in the garden and some people came out to help

Interviewer: Great and there are other additions?

Respondent 6: Since I got this sickness when I'm in with my peers they don't stigmatize me. My sickness doesn't fear crowds, my challenge is just when I'm about to sleep. When it comes to a gathering where we are going to cook, I joined them to do so. in the crowd, I can sit and relate with everybody, and nothing will happen to me but when it's time to sleep that's where the problem is. with the way my sickness reacts if I get at a sewing machine or trade or selling of bread (that can you give me money too) with the rest I can't go out of the sun

Interviewer: Great, let's continue

Interviewer: Please tell me about any health campaign activity that you involved your community.

Probe for names of the health campaign, when it was implemented, for how long it was implemented, what were the challenges, and what were the benefits.

Respondent 3: Yes, they gathered us to teach us that when someone this struck with the sickness, we should use cloth and tie the person down so that the head will not hit the ground because when the head hits the ground it can increase or give the person a different sickness.

Interviewer: OK any other

Respondent 1: Yes, Doctors have come here to encourage us and show us how to handle our sickness so that people will not stigmatize us in the community. They usually let us know how to keep ourselves. When your brother falls on the way and you ignore him and leave. It means you have stigmatized the person already. They made us know that the sickness does not transfer from one person to another. This was what the doctor's research showed them. The sickness is not transferable. So, when someone has this sickness because the doctors have educated us on it we have faith now. Ladies that are living with this sickness couldn't get married but because the doctors have educated us on it now, they are giving birth. The doctors showed us how to live with it and it does help us.

Interviewer: What were some of the unpleasant experiences you have trying to access health care as well as social protection avenues or opportunities? **Probe** for specific situations, when it happened, who was involved, etc.

Respondent 3: Someone called me a mad person and it hurts me I couldn't eat that day even though there was food, and it happened just in our community.

Respondent 2: there was a gathering, and I also went to the gathering it was in the hospital at ….. and they told me to go to the hospital, I went, and they checked me and gave me the pattern I should take my drugs, they said this drug should be taken ones every evening and they encourage me to keep taking it till am well. They picked someone in the hospital to write our names down, anytime it was time to take the drugs the person would go around and wake us up to give us the drugs, that was how it was till we left that place. When I go to the big hospital because there are a lot of people there who have come to seek medical attention when I inhale their perfume, it triggers my sickness, so they separate us into the room. Since then, I have not experienced sickness again.

Interviewer: Good has anyone also experienced the same?

Respondent 4: When my sickness is about to manifest and I'm on the way it will roll me off the way and I'll fall.

Respondent 1: (clarifies her by asking) If it happened at the hospital.

Respondent 4: I went to the hospital, and they gave me drugs to take, after taking the drugs I was sweating and later my body was calm, and we went home. When the sickness is about to manifest it will start by making my heartbeat very fast then it will put me to the ground. Now they have identified the sickness, and they give me drugs to cure it.

Interviewer: Good Any other

Respondent 1: My sickness is this way, in the hospital at times the sickness disturbs me till I visit the hospital. They admitted me when I got there and kept me on drips till, I urinated on the bed. The sickness is serious, but the doctors play with it. So that happened I wasn’t happy.

Interviewer: Good, let's proceed. What are the facilitators of SHGs activities?

Respondent 1: If I’m going to respond to this question. What we are going to be doing in this group that will make us move forward is peace and trust concerning work and hard work. If we put these first the group will be strong and move forward

Respondent 3: In the group if we can sit and consult each other the group will be able to move forward.

Respondent 2: I’m just there when am not feeling well and someone told me that if you don’t plant trees, you will be struggling that’s when I planted a cashew tree and added mongo trees too. Today when is dry season I make up 3 -4 bags of cashews and can sustain me for a while.

Respondent 3: When we get another NGO to come to our aid, it will help and make life easier for us.

Interviewer: Good we are moving forward. What are the barriers to SHGs activities?

Respondent 3: What prevents this group from moving forward is lack of work and hardship so because of that it does not make work move forward.

Respondent 6: What will make the group move forward is unity. When there is unity, we will go high. The lack of unity will not make the group move forward. Hardship too will not let the group move forward. If we are united and there is hardship, we can't move forward.

Interviewer: OK any other or we should continue?

Respondent 4: What will make the group go forward is when you help, we the poor. Some of our members don't have anything doing and we want you people to encourage and support(funds) us to come for meetings. This would help our sickness reduce, so we can move forward.

Interviewer: What can be done to address the challenges people with mental health conditions face in this community?

Respondent 3: To me, if could get an NGO that would come and help us with something, it would change a lot.

Respondent 1: With this sickness and struggle, we are crying to you (basic needs) people that Bugiya has a small hospital if the government observes and sees that we have a doctor in this small hospital. When the sickness is about to disturb the doctor can come to our aid. If the government can help us with that it will reduce our struggle. The clinic would help us because everyone is crying there is no money. Where there is sickness and struggle, money is needed.

Interviewer: What role do you expect government agencies ***to*** play in meeting the health and social needs of people with mental health conditions?

Respondent 3: To me if He could get us a hospital it would help in emergency cases either evening or morning.

Respondent 2: If He can get us the drugs, we have a hospital where we can keep it there so that if anyone needs it you can get it there. There are times you would go to buy the drugs, and you won't get some and when you come home without it(drug) you can't sleep.

Interviewer: Good is someone having a different opinion?

Respondent 3: Transportation to the hospital is a challenge for some of us. To get even a bicycle to go for drugs in the hospital is a challenge.

Interviewer: Good, let's move to our last question

Interviewer: What are your SHGs doing to ensure sustainability? What will you do without support from NGOs such as BasicNeeds Ghana?

Respondent 3: What we will do is, if the group can still maintain meetings and be able to discuss and contribute something(money) down.

Respondent 1: Looking at BasicNeeds, if they are going to take off their support. Even duo we are sick we have students among us. If they can help our student, some of our students are graduates (JHS, SHS) and needs support to continue. If they can help us with that and now leave the students they helped can now take care of us.

Interviewer: Good is someone having a different opinion?

Respondent 2: If they don't help us again, our garden, they gave us some skills about it. We have knowledge in that sector that is helping us get something to feed on.

Interviewer: So that's what you would depend on for survival?

Respondent 2: Yes, if we agree and continue with the garden and there is fertilizer or fertilized land. We can continue with the garden.

Interviewer: Any other recommendation for effective SHGs operations?

Respondent: We want animals to rear

Respondent: If we get Goats, Fowls, Sheep, etc. to rear. It can help sustain us.

Interviewer: Mommy it seems you have something to say

Respondent 4: If you can do that when the sickness strikes, we can use the animals to make some money for our treatment.

Respondent 1: What I will encourage is some of us are traders so if you give them animals to rear it will be hard for them since they don't sit in the house, so I will say they should give us money to do trade or better still they should give traders money and give those who want animals to rear animals. They should help everyone according to their need.

FOCUS GROUP DEMOGRAPHIC DATA

Interviewer: My name is…… and these are my colleagues…….. We work for ………. through Basic Needs. Today we would like to have a conversation with you all. We are very interested in learning about the operations of SHGs for people with mental health conditions and disabilities. We will only share the information we learn today in a general way that does not reveal the identity of anyone in the group. (As a result, respondents were giving numbers to conceal identity). With your permission, I will be recording our conversation. It's important that the information shared in the group does not leave this group. So, we ask everyone not to share who was here or what was said of the group with others outside the group when you leave here. We really want to hear what you have to say and want you to feel comfortable in answering questions however you want to. There are no right or wrong answers.

Wumpini would be taking note to make sure that we don't miss what you have to say. This will help us later when we go back and organize all the information that was shared today. The group discussion should last for 30minutes.

Interviewer: How do your SHGs operate in this community (ies) operate?

Respondent 1: Truly, our group is very supportive of one another, the help is that when one has an occasion the group goes to

know how the occasion is. Occasions such as someone has a naming ceremony, or funeral.

Interviewer: Great, we would want to know days/times of meeting,

Respondent 1: We meet on the first Saturday of every month.

Interviewer: How many would your membership be?

Respondent 1: Both the sick and care givers put together; we are over 60 people.

Interviewer: How are resources sourced?

Respondent 1: In terms of resources, BasicNeeds Ghana gave us chairs and canopies so when we have a meeting we use it. But in case someone (no member) has a naming ceremony or wedding and needs our chairs or canopy, we rent it out to them and make some money for our group.

Interviewer: Anyone with something to add?

Respondent 5: What he said is true, this is number 5 speaking. When one of us has a need of it and comes to request for it, we give it out. But if it someone comes to hire it, the money paid is kept so when one among us has a challenge we use that money to support that person.

Interviewer: Do your members attend meetings very well?

Respondent 5: By God's grace and power, they come.

Respondent 4: Truly, our group is very good. Anytime its first Saturday, everyone usually comes out in their numbers for meeting. Truly the chairs were donated to us, and I have even used them for a naming ceremony.

Interviewer: Which people gave you those chairs?

Respondent 4: BasicNeeds Ghana, I find it difficult to pronounce the name (laughs). When I had a naming ceremony, I was supported with the chairs because I didn't pay for it. So our group is so helpful to us.

Interviewer: Describe your day as someone with mental health condition or disability?

Respondent 2: Truly, as for me, this is number 2 speaking. When day breaks, the only thing I do is lying down. When it is time for salat, I go to pray and come back to lie down again. I don't have any business. To me, I have no business doing.

Respondent 3: When day breaks, I have a small business I operate. I sell provisions. After dawn prayer, I come to open the store to sell a little and I close around 11am.

Respondent 6: This is number 6 speaking. I'm a welder. I work from home. The little I earn is what I use to take care of myself, my health and everything that concerns my life. I leave for work by 7:30am. Everything is going well at my work site. I close from work by 6pm. This is what I must add concerning my work and how my day goes. May God bless us.

Respondent 1: I am a bicycle fitter. Some mornings, there could be an occasion such as naming ceremonies, or weddings at home, as a result, I may not be able to leave home early, sometimes I leave home around 9am and get back around 6pm that's how my day goes. thus, times I can leave the house around 7am thus I don't really have a specific time I leave home for work. When the day breaks, sometimes issues at home can delay the time I want to leave for work, so I really don't have a specific time I leave for work.

Interviewer: What are some the pleasant experiences you have had as someone with a mental health condition or disability?

Respondent 3: BasicNeeds came to support me I was very happy. I have six children and BasicNeeds came to support me with five hundred Ghana cedis so it could help to be getting some porridge for my children. That day I was very happy because I didn't know what to do and they came to give me such an amount of money, I was so happy.

Respondent 5: Truly, what BasicNeeds have done for us we wouldn't be able to say all except God. They gave me support and that support kept me till date, and I thank them so much. They called me and supported me with two thousand Ghanaian cedis that I should take the money take care of my daughter to learn tailoring and the education of the other siblings. I went for the support and I'm thanking and praising BasicNeeds Ghana so much.

Interviewer: Anyone with something to add?

Respondent 4: What they are saying, I have only heard but I have not seen it. I didn't have any support. I wouldn't lie. As I have not had support, what is it, it is because when we meet I only hear of it but I have not seen it. Even in …… we met.

Interviewer: Not only BasicNeeds Ghana, haven't you had any pleasant experience in this community?

Respondent 4: oh ok, I have had, I have had

Interviewer: And what is that experience?

Respondent 4: My friend ever did something to me and I was so happy.

Interviewer: What did he do for you?

Respondent 4: She gave me food. She gave me four bowls of maize. That I should use to feed my children because I don't have much to give them. When it finished, I went back, and she added me more maize and I came back to take care of the children as their father is no more alive.

Interviewer: Anyone with something to add?

Respondent 2: Mr. Sandoo and co came to take me out of ignorance into enlightenment. What I mean is that, how to eat was a challenge, what to wear was another challenge, going into the public was also another challenge. Why I say this is that they came to assist us with animals. So, the animals, I was rearing them. Because I have been falling it affected my brain, I went to the hospital, and they said that my heart has been injured and so they were going to give me medication. And the drugs, each tablet would cost me ten Ghanaian cedis. I was rearing the goats, and they were there. My father, the only thing he does is farming so he doesn't really care about me unless I am the one who tells him that I am sick. So when it comes to buying the drugs, I usually talk to chairman telling him how my condition is thus I want to pick one goat to sell and go to hospital and then he would say, "Organiser you can pick. The support they gave you is for your health so pick one of the goats to go take care of your health." So to me they took me from darkness into light.

Interviewer: Moving forward, what are some of the unpleasant experiences you have had as someone with a mental health condition or disability?

Respondent 4: Someone can just see you and stigmatise you. Are you getting me?

Someone would just see you and stigmatise you or sometimes when you eat food and there is left over, they would ask you to pour it away. That you should pour it away. Or someone would meet you on the road and look at you in a very demeaning way because of sickness meanwhile you are older than such a person. May God help you to help us treat our sickness.

Respondent 2: As number 4 said, truly when I sat among my friends they called me mad person. This number 2 speaking. Whenever I sat with my friends, they called me mad because I don't talk. I always sit quietly among them. I met Mr. ….. and co. When I was to join the meeting, the women leader took me to chairman and chairman and the women leader took me to the hospital. It was there that I got to know about the meeting and began going to take medications. Each time I went, they would give me medication for a month, other times for two months, other times four months, and I was taking the medication. As I take the drugs, one day I went to the hospital, and they told me that I should pay ten Ghanaian cedis. They said the drugs is no longer free, that when you come to take the drugs for free, you must pay ten cedis for them to write a note for you so that you would be able to come for the drugs for free. So, I told them that if I had ten cedis I wouldn't have even come to them. The one who is head of the unit at the hospital began shouting at me that what I have said is nonsense.

Interviewer: That was your unpleasant experience?

Respondent 2: Yes, I was very displeased and went home.

Interviewer: Anyone with something to add?

Respondent 6: Truly speaking, I have seen life and how everything is going and wish to bring this matter before my father because my father didn't know about it. There were times when I want to go to my work place, it was at work place they saw how I lived and told me that for me, I'm such a weak person and that they don't know what is wrong with me. I told them what was happening to me in my life, and they didn't believe it. There were times we were to work, they would be looking at me a way saying, "So-and-so why are you like that?" So, I informed them about my condition then I came home to inform my father. My father took me to the hospital. I began taking the medications and saw changes in my condition. That was when I realized how the drugs can bring a lot of relieve. That is what I have to say.

Respondent 3: Our sickness has a lot of stigmatizations. Even in my home, I have been stigmatised. Sometimes when I cooked food, no one would eat the food because of my sickness. Even at times water in my pot, people didn't want to drink it. They think they can get sick if they drink the water. Because of this, I went back to my father's house. Now I live in my father's house because of sickness and its attendant stigmatization.

Respondent 5: Truly, the sickness has a lot of stigmatizations to it. Even though I am number 5, in my house when day breaks, my children with me are two. It is only the children and I who converse in the house. My siblings and their wives don't converse with me not because of anything but the sickness. For the sickness I thank God a lot concerning the way they stigmatise me because of the sickness. Even in the mosques people don't want to stand by you. In my house when there is an issue, it outside I would later hear it and

no one would inform me because of the sickness.

Interviewer: That is what hasn't pleased you?

Respondent 5: That is what has displeased me. As a human being, you know your beginning, but you don't know your end.

Interviewer: We would like to know your source of funding, funding stakeholders, frequency of receiving support and the last time support was received and by whom?

Respondent 3: The last support we received was from BasicNeeds Ghana. They called us together just as we've gathered for this meeting and gave some cash support. They have also been coming out to support us with chairs and canopies and we rent them out. I am the treasurer. We rent out the chairs and canopies. They are those who have been giving us support. Anytime support is to come to us, it's mostly from BasicNeeds Ghana.

Interviewer: Does anyone have something to add?

Respondent 1: Where we get our funding from there are many yet not many because we still need more support. BasicNeeds Ghana has been supporting us, Social Welfare, and the Metro Assembly. Last year, they supported some of us with cash, some had thousand Ghanaian cedis, others had thousand five hundred cedis. In our group, they supported up to eight people. Those I know and can point out. It was BasicNeeds Ghana who collaborated and linked the group to social welfare and the assembly for that support to have been given. This is what I have to do concerning our source of funding.

Respondent 4: This is number 4 speaking, last year, they showed us kindness. When I say they showed us kindness, you know I'm an old person…

Interviewer: Which people?

Respondent 4: What is their name again, that is the assembly people. They called us and we wrote applications to them and the support came and we went. They gave us something small. As they gave us a small support, I'm an elderly person and not tough. If I keep the money, people will come and borrow the money and at the end, the money would be wasted. So, I gave out my money to be used to buy two animals. They were sheep. I took care of them, and they multiplied to four. One day they ate grass sprayed with poison and two died and left two. Recently, one of the sheep went and chewed rice and also died. Now it's left with one. It also got pregnant, and the baby died. Whatever the support is, it is also good. I thank God, when the one left populates, I can use it to take care of my health. If you have no money today and you are sick, you will amount to nothing.

Interviewer: What I want to clarify before we move on is from whom did you receive your last support?

Respondent 3: Our last support came from Metro Assembly. It was the assembly who recently gave us support.

Interviewer: You can't remember the year and month?

Respondent 3: It should be up to a year and a half. Metro Assembly called us, I got thousand five hundred Ghanaian cedis.

Interviewer: very good. In number 1's submission, he mentioned something related to our next question. Yet I would want you to throw more light on it. I would like to know your collaborations with government

institutions (district/municipal assembly, social welfare department and national health insurance scheme)

Respondent 1: We, BasicNeeds Ghana, social welfare, and metro assembly have met. We met for a discussion and came to some agreements. If I'm not mistaken it should be up to a year now. When we had our last meeting, the agreement was what the metro assembly would do to ensure they are aware of us so that anytime we come to them for support, they would be in a position to support us well and with enthusiasm. In that meeting, DCE was there, the head of social welfare was there. The name I can remember, Madam …. was there. Also, there was also a police representative, and I don't know which other NGOs, but they also had representative and the meeting was held. We met at the metro assembly.

Respondent 3: When they called us for the meeting, this is number 3 speaking. When they called us for the meeting, a nurse from the Tamale Teaching Hospital (TTH) was there. DCE was also there. Madam Hawa was there. The Director for Health Insurance was there. The health insurance director told us that anytime our health insurance expires, we should put them together and take them to Madam Hawa who is in charge of social welfare and she would bring for them to renew it free for us. So when we met, they told us that thus anytime our health insurance expires, when you send it to the social welfare director, Madam Hawa, she would take it to the health insurance office and they would renew for you. When we met them, they told us all this.

Interviewer: Please tell me about the kinds of training you have received to support you financially.

Respondent 5: Truly, some people have ever given training which would help us to support ourselves financially. Those who gave us that training was Gupkatimali and social welfare. They met us to talk to us. They were to give us animals to rear. They taught us how to take care of the animals so that we can benefit from the support. Because in these times, no one has anything in their rooms for the rainy day but if God allows and they give us the animals, and we keep them well and God helps us and the animals populate, the animals will be of immense benefit to us so they gave us. When they gave, some people's animals died. Even I, they gave me two goats, but they all died. When our animals died, others were fortunate, and their animals did not die till today. They are still keeping the animals and it's helping them but some of us no longer have any animal.

Respondent 1: What I can remember is that BasicNeeds Ghana contracted NORSAC to train us in business. In the training, they told us that when you have capital to do business, you don't lend the money out to anyone who just comes to borrow from you, especially family members. Most of them won't pay and that can collapse your business. Others just come to borrow from you because they want to collapse your business. They also trained us in the area of reproduction. If it's a woman, how to take care of themselves during menstruation. How to put on menstrual pad. All these, we were trained on it.

Interviewer: Describe to me the kinds of skills/competencies you will need to effectively support your operations as SHGs.

Respondent 4: As for the skills unless you come to train us.

Interviewer: Mention the skill you would need.

Respondent 4: As for me I process rice to sell. So I would prefer more training in that regard. That is what I sell. I sit in Aboabo market, Tamale, that the area of competencies I would need. That's the end of my contribution.

Respondent 5: What she said is true, truly I'm not well but I grew processing and selling the rice. I sell at the market, but I have no finances it makes it difficult for the business to grow. So, if you had to give skills training in the rice processing it would have helped me a lot.

Interviewer: So, what the things you would for effective training?

Respondent 5: The things we would need is money. That's our main problem, the money. It is not there.

Respondent 4: What she has said is true. If you don't have money, today, how much is a bag of unprocessed rice? How about a bowl? One bowl of unprocessed rice is twenty-five Ghanaian cedis. If you are to buy ten bowls, that's two hundred fifty Ghanaian cedis. We need financial support so please help us.

Interviewer: Any other thing?

Respondent 3: The skill I would want you to support train us in is that you know some of us are now old and we didn't learn sewing, weaving, and the others. It just the petty trade you have learnt. For the business, I sell provision store and others for me and my children to survive on. But it needs money. If you could support us financially, we could use it to trade so some profit could be generated for us to survive on it with our children.

Respondent 1: You are saying the skills we think would be of utmost help to us for effective operations of SHGs. Among us, there are young men and young ladies. First, weaving, when you train us in that skill, it would help the SHGs. In the radio and television, if you find a way so we could come on air, we the sick and care givers to educate the public on this sickness. It would help a lot. When we talk about the fact that sickness is not transmittable, those who are usually afraid and when someone gets seizures around them, and they run away and leave the person. As everyone gets to know the sickness isn't transmittable, when someone gets seizures in public people would come around to assist them rather than run away. All these would be of immense help to us. That's what I have to add.

Respondent 6: I would need training in farming or business or any skills training. This would help to make life go on well. With the farming, every year would need support for the farming season. The support is money. When we get money, we would be able to farm, if we get farm implements or farm chemicals or any other knowledge, we would need in the farming sector. We would use all these in our farms. By god's grace, when we get a bumper harvest, it would be of great help to us and our families. This is what I must add with regards to the skills training.

Interviewer: How do you involve the community in the operations of SHGs activities?

Respondent 2: This is number two speaking, the people we usually invite are Mr….. and co.

Interviewer: Here we want to know the people you involve and engage within your community.

Respondent 2: The person we usually invite is the assembly member.

Interviewer: What did you invite him for?

Respondent 2: We have ever invited him to come and teach us how to live within our community peacefully so that our lives would be better for us.

Interviewer: anyone with something to add?

Respondent 3: Our SHG has ever invited our Member of Parliament, Lawyer Haruna. He couldn't come himself, but he sent a representative to the meeting. We wanted him to come and know what we do because if you have a group and you are people who aren't well, it is good your leaders are made aware of you. As we are there, we've ever paid a courtesy call on the Chief of Lamashegu. So the Chief of ….. knows about us. Lawyer Haruna sent a representative to visit to know about our SHG activities.

Respondent 1: What I am to add is that, in our community, how we involve them in our activities, as the community knows this is a group of people with mental health condition and disability, so anytime someone gets this sickness, they quickly run to us so we can help the person to access medical help. For instance, if someone gets epilepsy, because they are aware of us in the community, they quickly run to us for help. They ask us what they can do to access medical health either for themselves or relatives so they can get well. We provide such guidance. When there is a need to send the person to hospital, we do so. In our community that's how we are involved in the community.

Interviewer: Please tell me about any health campaign activity that you involved the community.

Respondent 5: Yes, we've ever had a health campaign to talk to them. We told them how to keep themselves to prevent this type of condition. We also talked about the things they might do that can cause this condition which they don't know. For instance, there are some people, you know one cause of this condition is the way we beat our children. Some can be so heartless that they can beat a child to the extent of even hitting the child's head with a wall or any metal around. All these can cause this condition and some of us are victims of this.

Interviewer: So that was your message in the campaign?

Respondent 5: Yes that was what we taught so that it would to protect them

Respondent 4: This number 4 speaking. May God have mercy on us. Do you see what happened, only God can lead us in the right path. No human being has the knowledge. There is no human being who can know things like this exist in this life. May the Lord have mercy on us and show us the way to cure our sickness. This sickness worries us a lot. If you have it, you can't go into the public domain. No matter how well you keep yourself, people would still stigmatise you. You go to stand with someone, they would be murmuring and asking that why this person is epileptic and you're standing with them. Meanwhile, you didn't buy the sickness. It is the workings of God. May the Lord help and this sickness would leave our bodies.

Interviewer: What want to know about any health campaign you've had in your community.

Respondent 1: Last year, we gathered. We went to the ……. Chief's Palace. The Chief sent out the "gongong" beater to summon the

community and they gathered. BasicNeeds Ghana sent representatives. They call it durbar. They spoke to the people not to discriminate against us, they shouldn't stigmatise.

Interviewer: What were the challenges?

Respondent 3: When we were to have the campaign, BasicNeeds came and supported us because you can't go to a Chief's Palace without something to offer. They gave support so we could pay a courtesy call on the Nakpanzoo Chief. We spoke with him to gather his people and we talked to the people. That day, BasicNeeds even came with the drugs. We told them that this sickness is not transmittable so anyone who has it, they should do well so that there would not be discrimination and stigmatization. The Nakpanzoo Chief also added his voice to talk to the people. We gathered the people to educate them just as they have also been educating us. We take it home to also educate our community.

Interviewer: What were some unpleasant experiences you have trying to access health care as well as social protection avenues or opportunities?

Respondent 4: Truly speaking, as for me when I go to hospital, they don't want to mingle with me. They know it isn't transmittable. As for drugs, they give me drugs when I go. There are some of the hospitals they don't treat us well (at the end her utterance she mentions the Tamale Central Hospital) but for the clinics, they are very patient with us, encourages us to always come back. They don't usually deal with us in anger.

Respondent 6: I have ever been to a hospital and told them my condition. They gave me medication. I came home and took the drugs. It began to have effect on me. As number five said, you come home and get some unusual seizures and people look at you in a way you are not comfortable. When I go back to the hospital to tell them that the drug they gave me is having negative effect on me, they would change me a drug. They would change a drug and ask me to take that drug and there would be changes. I think that is what I also have to add.

Respondent 5: For me only one person ever insulted me in the hospital. I wasn't happy with the insult. She was a nurse. I wasn't happy so I drew her attention to it. I told her that what she has said to me is because of sickness she had the chance to say such things to me. For the sickness, she couldn't have said such nonsense to me. So when they are doing their work, they should be mindful that there is a future. I went to take the drugs. The drug was there but she told me the drug was not there. The doctor had not yet come. I told her to write it for me. She retorted is it not a written note I already came with. I should use that note to go buy the drug. I said she shouldn't have spoken to me like that. At the end of each month, when we come, they usually write it down, so they know so-and-so person has come. She shouted I shouldn't disturb her. I said to her to remember there is tomorrow. Truly what she told I wasn't happy.

Respondent 3: We have a particular nurse. This is number 3 speaking. We have a particular nurse. Even our contacts are with her. In case she gives you the drug and you have negative side effects; you can call to inform her. The next time you go, she would change your medication. There was a time they gave me a particular drug, the drug was too strong for me. I called to inform her and

she said I should return the drug. I retuned the drug and she changed me a different drug. So, the hospitals when you go, frankly speaking, when you meet someone who is sensible, they always encourage you and doesn't want to say anything to offend you. This our sickness doesn't want us to become unhappy. When they say something that can affect your mood, you can get seizures. They must keep encouraging us till we get well.

Interviewer: Number 3's response has taken us into our next question. What were some of the pleasant experiences you have had trying to access health care as well as social protection avenues or opportunities?

Respondent 2: Where a doctor spoke to me, and I was so happy was at the Tamale Teaching Hospital. I went because I someone informed me a new doctor had come to look after people suffering from epilepsy and mental disability. A friend gave me the information and I went to the hospital. When I entered, he bought Don Simon fruit drink for me. Gave me a sit to sit. He said I should make myself comfortable, finish the drink before I can tell him what is wrong with me. He said I should relax myself and eat the food. I ate the food. When I finished eating the food I was so happy for the treat. I told him I have been going to hospitals, but his reception and hospitality has made me so happy. He again took me to where I would take the drug. What he did to me I felt like crying because he led me for everything to be done. And he even used his car to take me home. He added me money which I spent for up to a month.

Respondent 1: As for me, Dr. David Abdullai, may the Lord move his generations forward. In his clinic, this is number one speaking, I went, and they examined me. One day, I went there when I was to leave, he gave me honey. I enjoyed the honey so much. I was so happy.

Respondent 2: Let me add to the Dr. David Abdullai's story. This is number two speaking. As for Dr. Abdullai, may God bless his generations. As for that man, I have not seen any doctor like him in Tamale.

Respondent 3: Dr. David Abdullai, when he was alive, he received us and treated us for so long. Even food, every month when we went there, he would give us food stuffs and add you the medications. It is his absence that has made us to go to the hospitals to buy the drugs. When he was alive, when you go there, he would give you the drug and add your food stuffs.

Respondent 5: Truly, as for Dr. Abdullai, everyone is grateful to him and prays that God would bless his generations and raise others like him. Anytime you went to his clinic, you would take the drug for free, and he would add you food stuffs, and give you lorry fare or he would ask his driver to drop you home. He would tell you that when you take the drug, you should sleep for several hours and wake up at such a time. He would tell your family that they should take care of you. That they should be patient with you because it takes patience to take care of a patient. But shouting and barking at the person; the sick person wouldn't have peace of mind and therefore wouldn't leave normal.

Respondent 4: May God raise people like Dr. Abdullai. I used to take medications at his clinic and the distance became a challenge for me. He asked of the name of my house; my name and he said I shouldn't come for the medications again. He used to bring the drugs to my house. When he was to bring the drugs, he would mix with food. He would tell me to take two or one pill. I would take the drug. He

would then advise me to lie down quietly because the medicine doesn't like aggression. When you take the drug and sleep, unless 3pm in the evening before you wake up and your body would feel so fine. So may God have mercy on him. May God raise people like him.

Facilitators and Barriers

Interviewer: What are the facilitators of SHGs activities?

Respondent 1: To my opinion, if government had added we those with mental health condition and disability to the LEAP monthly allowance it would have helped us a lot to promote our activities.

Respondent 2: To me, as chairman has said, to my opinion, Mr. ……and team should make it a point every to send an officer to our meeting. It would have helped the meeting to go forward for us.

Respondent 3: To me, what would facilities the activities of the group is that as chairman already mentioned. If government adds us to the LEAP monthly allowance it would help a lot to ensure that the group moves forward all the time and not retrogress. If members know something small will come at the end of the month, we meet on the first Saturday of every month so if we are receiving this allowance, no one will have to call the other to come for meeting. All the time members would have been very active.

Respondent 5: What I have to add is as chairman already mentioned. Government should add us to the LEAP monthly allowance. Majority of us have nothing doing. When day breaks, most of us have nothing to do.

Respondent 6: I thank my colleagues for their contributions. May God help and promote this group. I think this just what I have to add.

Respondent 4: You know how life is now. If you want to stand from the ground but doesn't support yourself with your hand, can you get up? The hand that can support us is the government. He should help us for God's sake because we don't know where to turn for help. Social welfare should help us. Metro Assembly should help us for God's sake. We don't have to eat. They should help us for God's sake.

Interviewer: What are the barriers to SHGs activities?

Respondent 2: They said we should be making some contributions. Last month we sat and agreed on the contribution, but I think it wouldn't help. Even what to eat sometimes is a challenge so how do we get money for such contributions. To me I think it is going to serve as a barrier to the progress of the group.

Respondent 1: What I would add is that when we call for meeting and members don't come in their numbers it would not help the group to progress. Also, when complacency sets in either on the part of the leaders or the members, it can also affect the group and its activities.

Respondent 2: What I would add is that when there is no trust among members, it would be a barrier to the progress of the group. Aside mistrust, gossip.

Respondent 4: When you call for meeting and everyone comes out. Whatever you plan everyone supports it. The group would progress. But when you call for meeting and members don't come, the group wouldn't

move forward. It would weaken. But the good news is that we have never called for a meeting and members don't come in their numbers.

Respondent 5: What I think would be a barrier to our activities is if everyone wants to prove they are wise. In a group when everyone wants to show they are wise, if not a lot of prayers, such a group would surely collapse. The way we used to be as a group, we are no longer like that. When we say there is a meeting, everyone wants to prove they know it better. That way, it can collapse. I would want to plead with this group to stop such attitude.

Interviewer: What can be done to address the challenges people with mental health conditions and disability face in this community?

Respondent 5: For we those who aren't well in the community, to my opinion, what will help to mitigate our challenges is if we have businesses we are doing. It would have helped reduce the discrimination and stigmatization we face in the community. But when they see you doing nothing, they don't even add you to anything in the community.

Interviewer: What role do you expect government agencies to play in meeting the health and social needs of people with mental health conditions?

Respondent 3: What we want government to support us with is to add us to the LEAP monthly allowance he gives to the poor so we can also benefit from it. When we are taking the allowance, it would help us a lot.

Respondent 1: What I would add is what government can do to help this our group a lot is that he has to make sure the drugs are available in every hospital and clinic so that any hospital or clinic we go to you would get the drugs for your condition. It would have helped the group a lot.

Interviewer: What is your SHGs doing to ensure sustainability?

Respondent 1: When we no longer receive support from NGO, they have already trained and taught us on how to seek support from other sources aside them. They are the district assembly, MPs, DCEs, etc. They trained us on how to seek support from these people. That we should write to them. So when the NGOs no longer support us and we implement what they taught us, the group would be sustained.

Respondent 2: They also taught us that when a time come and they are no longer giving us support, as a group, we can support one another. They gave us that training.

Respondent 3: Our group, when we no longer get support from those who are currently supporting us, they told us a time is coming when they would not be supporting us again. It is when you're a child that you suck your mother's breast milk but when you grow, they stop you. When they no longer support us, the chairs and canopies they have given us, as we rent them out to generate some income, we can buy more and add so it becomes a source of income for us when they no longer support.

Respondent 5: BasicNeeds taught us that as they are supporting us when they no longer support us what we think about it. When they gave us that training, for me I told them that as they are training us, like the saying goes, when someone is cleaning your front, you also clean the back. If not, one day you would suffer for it. I told them when they no longer support us the knowledge we've gained

through the trainings, we would implement that knowledge to keep the group.

Interviewer: Any other recommendation for effective SHGs operations.

Respondent 5: What I want to recommend is that I'm encouraging this group that we should unite. When we are united, it would help us a lot as a group.

Respondent 3: I'm pleading with everyone to be patient with one another. We shouldn't listen to gossips. This would help to continually move the group forward.

Respondent 2: we shouldn't listen to what people are saying. We shouldn't broadcast our challenges outside. We shouldn't also speak badly of the group to people. They might misinterpret us, and it can affect the progress of the group.

Respondent 4: We need patience. If we have patience, we can be together. May God give us patience.

Interviewer: I thank everyone for your time to participate in this discussion. On behalf of BasicNeesd Ghana and …….., I want to express our heartfelt gratitude to you. May God grant us good health and give us long life. Thank you all.

TRANSCRIPTION FOR FOCUS GROUP DISCUSSION

Interviewer: My name is…….., I came with my friends. My friends are called …….. Basic Needs sent…….to conduct this Survey but due to work schedules, he has asked us to represent him. So, we are representing ……. We have a few questions to ask. Please, if you have the answer, you can mention your number before you talk. Now we are zooming into the questions. I will ask the questions in English and translate them to Dagbani, if you can answer in English, good. If you can't, reply in your language (mampruli or moar).

SHGs Operational Structures Within Communities

Interviewer: How do your SHGs operate within your community (ies)?

Respondent 4: Our SHG, how we operate, we meet once every month to advise one another for the progress of the group.

Respondent 1: What we also do in our SHG is that we visit those who are not able to come for the meeting.

Respondent 3: As we sit for the meeting, we learn a lot so that we can take care of ourselves at home. The bad things we could have fallen into we are able to avoid.

Respondent 2: As we meet, we also learn how to take care of ourselves, we are also able to encourage one another, and can take care of ourselves better

Respondent 6: Sometimes the sick person may say they want a particular type of food by force, you must plead with them until they understand you so everything will be peaceful that's what I also have to add.

Respondent 5: I may want a type of meal. If I can cook that meal, I cook for them to eat but if they don't want anything you don't cook for them.

Interviewer: Describe your typical day as someone with a mental health condition or disability to me.

Respondent 5: I can wake up and doesn't want to talk, don't talk to me or that's not it?

Interviewer: (**Interviewer** clarifies more on probes) How do you begin your work when day breaks from morning, how does your day go? **Interviewer** goes over probes (*for time work begins and ends, role (s) played, number of days on work (if any)*

Respondent 2: As a person with such a condition in your home, you cannot leave the home as early as everyone, you must be a little patient and delay to take care of your needs before leaving home. And when you're at work and it's time around 3pm – 4pm you have to go home early to take care your needs.

Interviewer: Does anyone have something to add?

Respondent 4: when I'm about leaving home for work, I am a sick person so in the morning, I have to check how they are faring first. I leave around 8am, because of the sickness I cannot keep

long in the farm because being alone could be risky and dangerous, so I come back home from the farm by 3pm.

Interviewer: What are some of the pleasant experiences you have had as someone with a mental health condition or disability? (Translates into the local language)

Respondents: all ask for further clarification to the question

Interviewer: (throws more light on the question with the help of probes)

Respondent 2: What I will say as a pleasant experience is that I have had in this SHG is that when I come out and I don't feel like mingling with people but others want to so when I wants to mingle with people don't prevent me and don't me feel as I am no human. If there is a need to prevent me from mingling with a particular group of people, be patient explain to me and not make me feel it's because of the sickness, in that situation, I would be unhappy.

Interviewer: That's ok but you have deviated into a different question, this question has to do with a pleasant experience.

Respondent 3: The pleasant experience I would say is when I like a particular food and I eat it, I become happy, I have to get that food so that when I eats it I would be happy.

Respondent 1: When you give us the drugs the sickness is not able to disturb us much.

Respondent 4: Another good experience I see is that as you the sick takes the drugs, you become calm, as you are always hiding yourself and don't want to mingle with people and are able to chat with people for sometimes.

Interviewer: What are some of the unpleasant experiences you have had as someone with a mental health condition or disability?

Respondent 5: When people know you have this condition, they mock at you and do all manner of unpleasant things to you.

Respondent 6: There are some when they mock us, we begin to avoid the public and live in isolation

Respondent 4: what I also see which doesn't make me happy is that we like everybody but because of the sicknesses people especially those outside our families mock at our condition, saying we are 'falling-down', 'he is a mad person' and wouldn't want to come close to you that way it makes you angry and even the family.

Current Financial Operations of SHGs

Interviewer: Please tell me about operation of SHGs
What are your sources of funding?

Respondents 5: As we sit for this SHG meeting we do contributions to help the operations of the group. We contribute for a long period of time and when you do that, we use it to help one another that is when one of has a pressing need.

Interviewer: Which stakeholders have you been you been collaborating with to assist around your operations?

Respondents 2**:** It’s BasicNeeds Ghana who come here frequently to collaborate with us for the past few years in areas of training and financial support.

Interviewer: Are you able to get support all the time when you need it?

Respondents 6: Yes, we get support because our ‘susu’ helps but it’s not all the things we need that we are able to get.

Interviewer: When was the last time you had such support and who gave the support?

Respondents 1: It’s been two years since we had support, and it was from social welfare

Interviewer: Anyone with anything to add? (No response) Alright let’s move forward.

Interviewer: Collaborations with government institutions (district/municipal assembly, social welfare department and national health insurance scheme)

Please tell me about how you collaborate with governmental stakeholders in mental health care.

Respondent 4: We and government agencies are in talks. That is social welfare and the district assembly. We’ve written to them and asked for support from them. The letters have been acknowledged as received but the support hasn’t come yet. From time to time, we’ve had meetings with them to remind them about our request when the means becomes available they should remember us so we would be able to get drugs.

Respondent 1: We also work with Ghana health service we have a psychiatric nurse who takes care of us. Also, health insurance renews our cards for us.

Interviewer: When was the last time you had such collaboration, who participated and where was it held?

Respondent 1: Last year, 2022, we met at district assembly with the social welfare department and health insurance manager in the collaboration

Interviewer: Any other thing?

Interviewer: When you met what were some of the key points discussed?

Respondent 1: When we met, we agreed that when the district assembly common fund is paid by government, we would be given 3% of the common fund and the health insurance department also said they would be renewing our health insurance cards for us when they expire.

Interviewer: Has the health insurance been able to renew your cards?

Respondents: (Chorus) Yes!

Interviewer: Any other addition? (**Respondent**s says nothing)

Community Involvement

Interviewer: How do you involve the community in the operations of SHGs activities?

Respondent 5: As we contribute the ‘susu; money down and the way we assist one another it would motivate and encourage members of the community to want to join us.

Interviewer: Good, but I think you have not hit the question right on point as it demands, this would help you to understand better. For instance, when you are to engage someone, how are they consulted, is through written or phone call?

Respondent 4: This how we involve stakeholders and members to participate in our activities. There are some we go physically to invite; we sometimes invite the assembly member to come and have a talk with our members. We also invite doctors to come to us on how to take care of ourselves to the sickness doesn’t go beyond our ability to bear. Sometimes we go to talk to them personally other times we call them on phone or write letters to involve them.

Interviewer: When do you usually call them and when you call them what do you engage them to do for you?

Respondent 2: There are times we call them when we have a meeting, and we need support from them. So we request that they come so we could collaborate and plan the way forward for us.

Respondent 3: When they come, there are teachings they give us. They teach us that when you’re the sick person and realise your disorder is to affect you, and there is no medicine readily available, you quickly rush to the hospital so you can access drugs for yourself.

Interviewer: Please tell me about any health campaign activity that you involved the community.

Respondent 4: we’ve had the nurses come talk to us and the community about metal health issues and we also brought forth our challenges, and they provided guidance on how to keep the sick people and on how to seek assistance from the nurses and from government.

Interviewer: But have you had a meeting where you gathered the whole community to educate them on mental health.

Respondent 1: We have ever gathered the community to educate them on the need to stop stigmatisation against persons with mental health conditions and disability.

Interviewer: What were the benefits and challenges you are uncounted.

Respondent 4: The challenge we encountered was that when we spoke with the nurses about the drugs, they said government has not supplied them with drugs and if you are someone with frequent seizures and goes to hospital and you are told there is no drugs, do not think that the drugs

are there but they refuse to give you. So, you the patient would have to find out where drugs could be acquired so you go and buy so the nurses can treat your patient for you

Respondent 1: After we had the community health campaign, I have realised that there is a change. The rate of stigmatisation in the community has gone down drastically.

Interviewer: What were some of the unpleasant experiences you have trying to access health care as well as social protection avenues or opportunities?

Respondent 1: When we started and went to district assembly and ask for our 3% of the DCF they told us that people with mental health condition and disability were not part of the beneficiaries of the fund. It's rather the physically challenged, not mental health patients and that did not make us happy.

Respondent 4: Just as number 1 said, we sent our names for help, district assembly told us they only know of widows and the physically challenged. But I think we the sick are more important, but they refused but rather gave assistance to widows and the physically challenged that is what made us unhappy.

Respondent 2: When district assembly told us they only take care of widows and the blind and the physically challenged, we were sad. We are also people with challenges but why would they do selective assistance. It looked like they had no regard for our condition. We weren't happy.

Interviewer: Any other thing?

Respondent 3: I have ever been to hospital though it wasn't me, someone came with an epileptic who had seizures during other patients and people. When she fell everyone run away and left her I had to run and assist the care taker so we hold her and raise her. I fetched water for her to wash her up. This experience made me unhappy.

Facilitators and Barriers

Interviewer: What are the facilitators of SHGs activities?

Respondent 2: BasicNeeds Ghana have been visiting us to train us on how to take care of ourselves and our members. How we can leave together so that there would not be problems. When we have a challenge, how we can access support so that everyone can be happy.

Interviewer: Anyone with anything to add.

Respondent 4: When we started with BasicNeeds Ghana and they come to educate with us to train us how to take care of ourselves so that even when there is no longer support from anywhere, we should be able to seek for support for ourselves

Respondent 1; When covid came, BasicNeeds came to gather us to train us how to take care of ourselves to avoid contacting the diseases.

Interviewer: But among yourselves what are you doing to facilitate your activities?

Respondent 2: We are also doing on our own as number 5 earlier mentioned is the 'susu.' So we are able to contribute for the 'susu' so in case one has a challenge or someone's medication has

finished and they have no money to buy, we can go into our coffers to assist such fellow with money to buy and when they get the money, he brings back and we put back into the coffers and when another has similar challenge we do same.

Interviewer: What are the barriers to SHGs activities?

Respondent 2 Our area of challenge is when you have someone to go but the sick person isn't very fine whatever you wanted to do, you wouldn't be able to do, if you wanted to go somewhere, you would not be able to go in that case it would disturb since you are not free to do your work as you wished to.

Respondent 1: Another challenge is the drugs. We don't get the drugs as we wish to.

Respondent 5: Another challenge we face is that if they could come to build a hospital or something for easy access to health care.

Respondent 4: Other challenge I see is that we have our own nurses and doctors. I this area I think our mental health workers are too few. We have only one such nurse. If they are more psychiatric nurses, it would have helped a lot. But one person all the way from Bunkpurugu to Nasuan. So sometimes you would need a doctor to take care of your sick and sometimes you have bought your drugs and injections, but they wouldn't be one.

Interviewer: What can be done to address the challenges people with mental health conditions face in this community?

Respondent 4: what I think when it's done would help a lot is that we would want to plead with health directorate and government to send us more psychiatric health workers to our area so anytime we need we can easily access them faster. And also, our drugs should be made available and accessible in our various hospitals.

Interviewer: Anyone with something to add?

Respondent 3: I think if we could unite to get our leaders so we can all take our challenges to DCE and MP, informing them that these are our challenges, and we need their assistance. That we lack nurses and lack drugs. they should try to assist us I think that would help to reach the government faster.

Interviewer: If anyone has something to add say it before we move to our last question for this session.

Interviewer: What role do you expect government agencies to play in meeting the health and social needs of people with mental health conditions?

Respondent 2: what I think government can do to help us patients is that the drugs are not available in the hospitals. So, since the drugs aren't available in the hospitals, there times the sickness would be disturbing you and your medications are finished that's when you begin running around to get the drugs. So, if the government did her best to ensure that the drugs are available in our hospitals so when you have a problem and you run to the hospital you would get drugs.

Respondent 5: That is why I said before that if we had our own hospital so the medicine can be there so when you have seizures or your medications are finished, you can easily go for your medication at the hospital and go back home.

Interviewer: (clarifies) so you wish the government would build mental health condition and disability hospitals?

Respondent 5: Yes, yes!

Recommendations and Sustainability

Interviewer: What is your SHGs doing to ensure sustainability?

Respondent 4: what we are working on so that in case BasicNeeds Ghana ceases to give us support either today or tomorrow. We have started a 'susu' contribution. We would also want that as of now, there could be support given us in the form of livestock or birds so that we begin rearing so the day BasicNeeds would no longer give us support, we would be able to survive or such help could come from government so the day BasicNeeds can no longer support us we can depend on it and our susu to survive.

Respondent 1: It is also good we meet frequently.

Interviewer: Anything to add.

Respondent 2: As 1 mentioned about our meetings. If we are meeting frequently, the day Basic Needs would no longer support us, and we continue to meet to encourage one another it would help to sustain the group.

Interviewer: Any other recommendation for an effective SHGs operations.

Respondent 4: To me, I think as we meet frequently, we should agree so during the farming season, we can go for by-day farming work so we can get more funds to add to our coffers.

Respondent 6; Also, during groundnut harvesting, we can go to work to make money to assist the coffers.

Respondent 3: There are a lot of work I think if we have the energy and ability, we could also go for groundnut uprooting or maize harvesting. That is what I also think.

Respondent 4: I think that if we go introduce ourselves to the chief and he is aware about this category of people has a group in his community. Anytime we need help or support, he can go on our behalf to seek for support on our behalf. If we inform the chief and receive his blessings, it will help us.

Interviewer: We want to thank you all so much for your time, I would stand on behalf Dr……. and BasicNeeds Ghana to thank you so that lets continue to be united and may the Lord bless help us all.

FOCUS GROUP DISCUSSION

FOCUS GROUP DISCUSSION

Interviewers: Good morning to you all. My name is …….. My name is These are my colleagues BasicNeeds Ghana has sent Dr. …… to come and take this information from you and he has also sent us to stand on his behalf to take this information. We will not use your names, we'll give you numbers. When you are to respond to a question, mention your number and respond to the question. When I ask a question, any of you can raise your hand, mention your number, and give your response. When someone speaks and you have something to add, mention your number and add what you have to say. You would be number 1, my sister would be number 2, my uncle would be number 3, you would be number 4, then number 5 and number 6. I want us to move straight to our questions.

Interviewer: First, how does your SHGs operate within your community (ies)? (probes added)

Respondent 1: We started by meeting every Sunday without a miss, that was how we began. But we continued for a while, there were challenges, so we began meeting first Sunday of every month until we again realized members were forgetting our meeting days Even I myself was a victim and we met again and came to a conscientious that we would meet first and last week of every month so every month we meet twice.

Interviewer: Any addition

Respondent 5: As 1 said it's true, we used to forget the meeting days now we have rearranged it. And we come we greet one another to know how everyone is faring, check the number of people who came for the meeting, and then we sit to hold our meeting.

Interviewer: How are resources sourced for the group

Respondent 2: For resources, we make contributions of one 1 cedi. It can happen that your medications are finished, and you go to the hospital and they write for you to go and buy Even if your money isn't up you can borrow some money to go acquire some medications while you continue the 1 cedi contributions.

Interviewer: Any addition

Interviewer: Describe your typical day as someone with a mental health condition or disability to me.

Respondent 1: Based on my body condition, when day breaks…

Interviewer: (interjects) I mean your work routine in a day…

Respondent 1: Yes, I will get there, every morning based on my body condition I think about the work I would do concerning my health. I think that I can make money to keep myself so in case I have any challenge, I can use it to assist myself, that is my thinking.

Interviewer(s): What we want to know is when the day breaks, what you do from morning to evening, the time you go to work, the time you come back from work, etc. as someone with a mental health condition or disability.

Respondent 5: Because we are not well, we are not able to go to work as everyone. You can go to work and seizures can distract you and you wouldn't be able to work. Unless you are patient with yourself, work small for the day and then the day you are very strong you go to work.

Interviewer: 2 was to add something

Respondent 2 Sometimes when the day breaks, it is not every day you would be healthy and strong enough to go to work, the time you go to work, there are times there are people at our work sites who stigmatize us and discriminate against us, so something happens, they shout and call you mad person. This makes me feel relaxed to go to work but when you go to work and you are well received by your family, your mind would be at work and every morning you would be enthused to go to work. So, there is no peace of mind as you wouldn't have the desire to go to work.

Interviewer: Any other, Number 3 you are yet to speak. Ok, let's continue.

Interviewer: What are some of the pleasant experiences you have had as someone with a mental health condition or disability?

Respondent 1: It usually happens sometimes I can meet someone I've not seen for a while and He asks how am doing, He encourages me by telling me I will be fine. When that happens, I feel happy because someone cares for me, and I feel someone empathizes with me. So, it gives me joy and happiness knowing that I am considered part of society.

Respondent 4: Sometimes if I go to meetings am always comfortable because I can look left and right and know that this is my colleagues but if am in the house, I will feel lonely.

Interviewer: That's good. Number 6 you've not said anything yet or we should move forward. Who else has had a pleasant experience? Number 1 spoke, and Number 6 too. Now let me ask this question.

Interviewer: What are some of the unpleasant experiences you have had as someone with a mental health condition or disability?

Respondent 1: It often occurs that you will be among your peers to do something. When something happens, they make derogatory comments about you. When that happens, you will not be happy because you are not expecting people to stigmatize you in that manner. So, when that happens and you are at the place at that time is always a challenge to you because how bad am I that people don't want to relate with me?

Respondent 6: What they said is true because the conditions wouldn't allow us to live our normal lives, sometimes you travel to different communities because of our condition we get worried that you may get attacked, and if it eventually happens what you were there for you wouldn't be able to do it, after that all mind will be unstable and because of that you may have to return home, you may go with the condition to any community and before you arrive you get the attack, if its

mentally challenged you will struggle and those you were going to work with because of the experience they begin to stigmatize you, so is a lot of thinking for us,

Respondent 2: It may happen because of our condition, assuming they send you for an apprenticeship and it happens that the day you arrive, and you had the attack, everyone's attention comes on you even if you are given a task none comes close to you again and because of that you are not able to freely stay and learn the work.

Interviewer: is asking for more experiences

Respondent 5; It has never happened even you can go to the hospital to collect the medications and if the attack comes you will see the nurses take you into an isolated room until you regain your full consciousness.

Interviewer: Number 3 we are still expecting your contribution

Interviewer: Current financial operations of the group and our first question is where you get monies to sustain this group and where do you get them from,

Respondent 3; Truly as number 2 earlier contributed, when we attend meetings, we make contributions one, one cedi down If it happens that one of us has no money and his medications are finished, he should come and borrow, I think when we started this has been our source of money, but we do get help from NGOs as you have come.

The **Interviewer:** is asking for more clarity on which NGOs specifically came and the kind of support.

Respondent 3; like Gupkatimali has never come and given us help and basic needs Ghana has never come and given us support, this is where we get our support from.

Interviewer: I want to ask how the help comes, like Can you describe in a year how many times you receive them?

Respondent 4; The help we have been getting is not frequent it can take one year to four years at the time it comes occasionally.

Interviewer: so, can you remember the last support you had and from which organizations

Respondent 2: 2020, In the year 2020 we received support from basic needs Ghana, they came and helped us with tailors and sponsored our apprenticeship.

Interviewer; So, is that the last support? that is very good, does anybody have anything to add so I can continue with the questions?

Respondent one: after receiving that support in 2020,2021gupkatimali also brought support if only is permitted to mention it then they brought us foes dresses and some bags to we who are not well, they were small backs that you can give to your children to go to school among them were others like purse and some shirts even thou they weren't bad dresses, I think that was the last time basic needs Ghana gave us support that was in 2021.

Respondent 4: Last year that was 2022, we also celebrated our mental health day, and then we got some support from Songtaba to help us buy water for our clients.

Interviewer: that's very good let's continue, we going to discuss your Collaborations with government institutions (district/municipal assembly, social welfare department and national health insurance scheme)

Respondent 1: we had a meeting with the health insurance on that day we called the social welfare, the health insurance director, and the assembly the meeting took place at the health insurance, on that day the psychiatry nurse in charge of us was also present social welfare director was present, the insurance director was present. The one who came and we organized the meeting was Mr. ….. from Basic Needs Ghana. He brought to our knowledge that if we are not well that is we the clients, he told us that health insurance renews our cards free of charge or any new without the health insurance card they offer such members too help to acquire the card and they also told us we have some support from the district assembly, I think we have given assembly letters several times, they informed us that truly our support is with them so we should choose among our self's some for support, also we met and we mentioned our challenges which is our medications, we wrote letters to the district assembly and social welfare. just that we didn't get any help aside they are reassuring us, but this year helped one of us and encouraged us to be patient if we get to the coming year, they will see to it that we will receive their support that we are not the only group so we should bear with them in patience.

Interviewer: is asking for the last meeting month with them

Respondent 1: I think the last meeting we had where they informed us, they have finished the supports they have unless next year was two months ago this year. I went and told them within 2023 that I wanted to know because we had a challenge with them, they said that it is only the blind and the physically challenged that they want to give support. When I went there, I wanted to find out the number of us they said they would support because they said they would want to know our total number before they would be able to know the amount of support they could give us. So, we have met with them, and they said we want to know the number of us who haven't received support from them so they can plan how best to support them so this year we met with them.

Interviewer: Moving forward, how do you involve the community in the operations of SHGs activities?

Respondent 3: Yes, we involve the community in our activities. Anytime there is any gathering, and they need people to come we also get involved, for example, in clean-up exercises, we also get involved so we can ensure the cleanliness of our environment.

Respondent 4: when we were celebrating our mental health day, we invited the assemblymen who are the mouth to the community so they can also talk about the stigmatization.

Interviewer: Please tell me about any health campaign activity that you involved the community.

Respondent 1: Yes, on World Mental Health Day, we gathered a lot of people, as 4 said, we had the assembly members and community leaders.

Interviewer: What were the benefits and challenges you faced?

Respondent 1: We made the public know it seems they are discriminating against us and we the sick even in our families when something is happening, they talk to us in a way that doesn't make us happy because when you do something and they aren't supposed to say some things, they say them to you. We were able to educate them that we're a part of them, and whatever that is happening to us they are supposed to give us peace of mind thus we need their support even if they cannot give us financial support, they should be able to inform us about where we can get medical support. All these we educated the public.

Respondent 6: To add to what 1 said, in our zone when you go to register people with mental health challenges, because they are stigmatized, they are usually not willing to make their condition known.

Interviewer: We would want to know the challenges you encountered during the health campaign

Respondent 4: We also educated the people on how to come out because if you are hiding in the room and there is a problem we cannot help like sometimes someone would look at you because you are sick, they can impregnate you and leave you, that one we talked about it and the community leaders gave us a number that in case of any such issue, we should call and they would arrest those people because they are infringing on our rights.

Interviewer: Any other thing before we proceed

Respondent 3: As 6 said, sometimes it can happen because they stigmatize, some of them hide, and some families even lock them up in chains and rooms, during the campaign we made them to understand that we are also human beings, and when we come out to meet our colleagues, it gives us peace of mind but they are locked up, it makes them to think a lot but when they come out as we are out it would encourage a lot of them to own but most of them are not owning up.

Respondent 5: I think the reason they lock some of them up is because some of them can come out and run into cars, or gutters would be dangerous for their health that's why I think some people lock them up.

Interviewer: Have you had any unpleasant experiences while trying to access health care and social protection avenues or opportunities?

Respondent 1: Yes that happens, you can go to the hospital and some of the nurses can stigmatise you because of the epilepsy or mental disability. They won't give you the needed attention. But as for the psychiatric nurses, they are better. None of them has ever done us anything that makes me unhappy. For them, anytime you go they have time for you but for the other nurses, they can treat you in a way that makes you feel you are not a human being.

Interviewer: Anyone with such an experience also.

Respondent 4: Even the patients those who are sick and they come to the hospital, they also stigmatise saying that are you looking for your mental health nurse, go and look for them this not the place to look for them.

Interviewer: Any of with such an experience also?

Respondent 6: That is there, and it is everywhere. Because you have the sickness, you know, not everyone has commonsense. When you go to hospital some of the people would treat you any how and you look at your life and feel so sad, but it is God who has allowed that to happen to you. So, it exist.

Interviewer: have you also had any pleasant experience while accessing health care?

Respondent 5: There are pleasant experiences. I have been to the hospital here to take the medicine, and the nurse was very caring. The nurse asked of how I was faring and treated me kindly and I was very happy.

Interviewer: Any other person with such an experience?

Respondent 1: I have ever gone to take my medication. I came across a colleague who was brought from ……. When he came out to go home with the person who came with him, I was so happy the way the nurse received them and the caretaker was also so happy because the moment they came, the nurse said she has realized that as the patient is taking the drugs he has changed completely and, but he has added weight and what is the reason. He was mentally disabled, and the patient responded he has been eating a lot of eggs. That when he finds people eggs, he picks them and eat, and the nurse replied that we are supposed to eat one egg per day but when you eat too much it can have negative effects on you. And the patient replied, "Is that so?" then the nurse said yes. Then the nurse told the patient to take meat and eggs with moderation. When we came out the mentally disabled person said he likes the nurse but not taking plenty eggs and meat is tough. I was so happy the way the nursed received them.

Interviewer: What are facilitators of SHGs activities?

Respondent 4: Anytime we have our self-help group meeting, it is good the psychiatric nurse or mental health nurse to be there. Sometimes too it good for the social welfare to be there so that we will also feel like we are part of the world.

Respondent 5: When it comes to the facilitators, I think if we get livestock to rear it would also help to occupy us and keep us happy.

Respondent 6: What can also facilitate our activities is when we get skilled work, it would help us to have peace of mind, and you can go to the work every day so you can also be a part of life.

Interviewer: Anyone with any other ting to add?

Respondent 4: If we also get finance from other NGOs to start business because most of us are not working. So, we also start small business or rearing of animals

Interviewer: Number 3 anything to add?

Respondent 3: Ok to my opinion, those who come to visit and talk to us about our welfare, I think if they could get assistance for us. There some of us when we take the medication and we are fine, when you are fine but has no work to do, if we could get some financial support, we could use to buy toffee and biscuits to sell so that we will be able to get money from the business to feed because hunger is one thing that makes our sickness more serious.

Interviewer: Barriers to the activities of the group.

RESPONDENT 1: If it happens that we have a lot of petty quarrels or lack of concern by members. You did not see someone in a meeting, and you don't visit to know why they weren't at meeting this week. When you follow up on the person, and their relatives see that, these people came to check up on their colleague who wasn't at meeting to know what is wrong with them. Because of that we go for house-to-house visitations. We don't joke with it. We do it a lot. Even when our days of meeting is not up, still we visit our members so that they would know we have so much concern for one another

Respondent 3: What I would add, it can happen that you sit for a meeting and wants to come up with ideas that would help the group to move forward. Someone can come up with an idea and when that happens, and you want to say that what one person is saying cannot work but the other person's contribution doesn't make sense. It can make the person feel you are discriminating against them, or you are demeaning them. You must take both suggestions and say that what both have said is good and then look at the two ideas and see how best you could modify it to make it workable.

Interviewer: What role do you expect government agencies play in meeting the health and social needs of people with mental health condition?

Respondent 1: You have brought a very important issue up. That has been our major challenge. We need drugs. The drugs are our main issue we want government to address it so we could be getting the drugs and not this go-and-buy, go-and-buy. That each time we need the drugs government would help to ensure that it is available in the hospitals, so we go pick. All these would help to promote our health.

Secondly, we spoke of support as number 3 mentioned earlier, when day breaks and you are fine but has nothing to do it is also a sickness. So, we would need the drugs and support that can transform our livelihood.

Respondent 6: Another support we would need is that some of our zones when you go to our hospitals you wouldn't find them as we need. They don't care for us there. Even you could help to advocate for us so that we the patients would know that as we are there, they fight for us. In our zones that doesn't exist. So, if government could look at it, it would have been so helpful to us.

Respondent 3: What I also see that they should do for us is that our nurse is only one because he is only one, government could try its best to increase the number to two or three. So when someone isn't well and they cannot carry the patient to the hospital, because he is the only one, when he goes, no one is there again but when he goes and there is one more nurse left at the

hospital so the one who is also able to come to the hospital can receive treatment. So, if they could it.

Respondent 4: More to the point if a person relapses. It is good the person should get injection but sometimes it is only the drugs that is there and that one is not even at the clinic. You must go and buy. Sometimes you go to the drugs store, some would not be there. You must be there like that.

Respondent 6: Like madam said, it happened to me. This is number 6 speaking. When it happened to me one day, it was the police who did their best to assist me. They picked me and laid me at the police station for sometimes before my people came for me, but it isn't the job of the police. If they were there, wouldn't they have helped?

Interviewer: What is your SHGs doing to ensure sustainability?

Respondent 4: They educated us that we have a common fund at the district assembly. We should always apply every year or a year we should apply twice. We have been applying but we don't get anything. This year, only one person received support from them. That's the place they told us that's our mother place. We should always go there for any help that we want

Respondent 1: What we can do in addition is that those of us who have been given sewing machines, driers, and animals, when a time comes and we don't get support from anywhere, at that time, it would mean that we ourselves should come out to work on our own even if it means we contribute. For instance, if six of us are tailors, and four have livestock and five are hairdressers, when we meet, because of the unity, we can take a decision to be contributing money down so that anytime we have any problem we can use that money to sought it out. So if we are able to get support in these areas I have mentioned, because of our unity, we would be to sustain the group so that it can move forward.

Interviewer: Any other Recommendations for and effective SHGs operations.

Respondent 1: The way you have come is a very good thing for effective SHGs operations. They would know that not only we those who are not well are concern for one another but there are people elsewhere who have special concern for SHGs for its existence. When it even happens that we say visitors are coming to meet us, all these are ways that boost our moral and those who are not members would usually want to be part of the SHG.

Respondent 4: The education we receive from the NGOs is also building our efforts on the groups because it means that without those people the groups wouldn't have been existing.

Respondent 5: What I would add is that we should encourage one another to take the medications. When you go to the hospital to take the drugs, you have to take it.

Interviewer: We are most grateful for your time; may the Lord help us. When opportunity presents itself and we meet again, we will meet you in good health. Thank you so much.

(HAND CLAP)

FOCUS GROUP DISCUSSION

Interviewers: Good morning to you all. How is home? And your family? We thank God. My name is ……. We have come and take this information from you, and he has also sent us to stand on his behalf to take this information. We have questions for you but before I start asking the questions, we don't want anyone to mention or call his/her name. So, I'm going to give you numbers when you get up to answer the question you first of all mention your number and now answer. so, uncle, you are #1, grandfather you are #2, Mommy will be #3, the next number 4, Mommy you will be #5, and finally grandfather, you'll be #6. I hope we have understood what I said. If you can't pronounce your number in English just pronounce it in Dagbani, I hope you all understand. I also want to plea that when you are about to talk, please speak louder so that our recorders can pick it up.

Respondent: yes

Interviewer: OK so my first question is

Interviewer: How do your SHGs operate within your community (ies)? · **Probe** for times/days of operation, membership, convening of meetings, frequency, participation during meetings, how resources are sourced, etc.

Respondent 6: This is how we have our meetings; in a month we meet once and that is always at the end of the month the last Saturday or Sunday of the month. That is how we meet.

Interviewer: Any other addition

Respondent 2: This is how we usually meet; we meet twice every week and that is on Friday and Sunday.

Interviewer: OK any other addition

Interviewer: Describe your typical day as someone with a mental health condition or disability to me. ***Probe** for time work begins and ends, role (s) played, number of days on work (if any)*

Respondent 5: when day breaks it's not every day that you feel very well there are some days to be OK some days to you know be OK and so even if you have somewhere to go you can't go unless you are OK that's when you can move out and know whether the work you are doing you will be able to work today.

Interviewer: So now that you are OK what time do you always go to work and what work do you do?

Respondent 5: In the morning when I wake up at 6:00 am, I move out to set fire for my pancake business, if I'm feeling fine, I will sell for a while but if I'm not feeling fine, I'll close early.

Interviewer: Any other opinion?

Respondent 2: For me, my help is BasicNeeds, Gubkatimali, and district assembly they help me to farm. I farm groundnuts and maize. My work is just to farm groundnuts and maize.

Interviewer: OK so when the day breaks what time do you go? And what time do you come back?

Respondent 2: When the day breaks and I go to the farm, I close around 12 noon and now come home to rest so that I can go back the next day.

Respondent 3: For me, I don't have any trade. The only work I do is, go and help Nba Lawyer grind meals. That's where I am working. We are helping those who come to grind their meals so when they come, and we help them at the end of the day do get us something small so that we can share among ourselves and we are 8 in number Because we are eight sometimes I don't go early sometimes I go around 12 noon or 1:00 PM and I will be there till evening before I go home.

Interviewer: That’s good I'm happy with the way you are answering the questions. Now let's proceed.

Interviewer: What are some of the unpleasant experiences you have had as someone with a mental health condition or disability? How has being a member of SHGs contributed to this experience in this community?

Respondent 6: The unpleasant experience I had was on the day of voting, everyone was in the queue, and I came and joined. I was in the queue when my sickness was triggered, everyone ran and left me alone over there and when I woke up, I realized I was alone over there I really felt bad so, I left the place to go to the house.

Respondent 2: The most unpleasant experience I ever had was when BasicNeeds gathered us for a meeting to give us drugs here in this town. I came to the meeting and fell to the ground because I was dizzy and they took me to the Buntanga hospital, that's the most unpleasant experience I ever had.

Respondent 1: For me, it was in school, It was Friday and we were in class and the sickness got triggered All my classmates ran out and left me. I really felt bad that day and that was what took me out of school up to now I have not gone back. I now went and learned a skill. I wasn't happy about it at all.

Respondent 4: I went to play football, and I was playing with my friends when the sickness got triggered, I was down, the time I regained consciousness I didn't see any of my friends again.

Interviewer: That’s good I'm happy with the way you are answering the questions. Now let's proceed.

Interviewer: What are some of the pleasant experiences you have had as someone with a mental health condition or disability? How has being a member of SHGs contributed to this experience in this community?

Respondent 6: What made me very happy was, in 2015 that time the DC for this district helped us with some money which was 5000 cedi, and it was used to buy medicine for our office. Also, Gubkatimali and BasicNeeds Ghana also supported us with some money, and we used it to develop ourselves. Up to now, we are still benefiting from it.

Respondent 2: What made me happy was when our DC called us and gave us 4 sheep each to rear. It made me happy. BasicNeeds and Gubkatimali also called me and gave me something small and that was 6 (six) years ago they supported us in farming. That was what made me happy.

Respondent: What made me happy was that Gubkatimali and BasicNeeds came and gave us clothes.

Interviewer: Can you me the year or the period

Respondent: That would be like 4 years ago. They gave us money before they came back to give us clothes. It was during that period we were excited. And since then, we haven't heard anything again.

Respondent 1: BasicNeeds and Gubkatimali came and gave us clothes and it made me happy, they also gave us 400 cedis to buy life stock, after buying and rearing them they have now multiplied. This is what made me happy.

Interviewer: Great, May God let it multiply more. Amen. Now let's continue.

Interviewer: Please tell me about the operation of SHGs

·**Probe**: sources of funds, funding stakeholders, frequency of receiving support, last time support of received, and by whom.

Respondent 5: Is not all the time we get funds or support, but it comes periodically Nonetheless BasicNeeds came and gave us 300 cedis each and it benefitted us well.

Interviewer: Can you tell me the year or the period?

Respondent 5: For about 5 years now

Respondent 6: Where we usually get support from is, sometimes within this group. We usually contribute money any time we meet and it has helped me, I have people here who can testify that when BasicNeeds came and gave us COVID-19 drugs and preventive stuff (Nose mask, hand sanitizer) and it got finished we used the money we had in our account to continue buying the sanitizers and the hand wash, so anytime we have met before we sit for the meeting we wash our hands first.

Respondent 2: We also make small contributions in our group. We received support from BasicNeeds too. Sungtaba also came and gave us some support.

Interviewer: What support did you get from Sungtaba?

Respondent 2: They come every 3 months and when they come, they give us an amount of money for 3 months before they come again.

Interviewer: Please let's all bring our focus here so that we can answer the questions well. Now let's continue.

Interviewer: Collaborations with government institutions (district/municipal assembly, social welfare department, and national health insurance scheme)

Respondent 6: District assembly is those we usually collaborate with, I remember 2-3 months ago our doctor called me to help social welfare fill out some documents with our details, They said they were going to send them to their office, but we haven't heard anything from them again even duo we keep in touch.

Interviewer: Which period was that?

Respondent 6: 2 months ago, or may not even reach 2 months.

Interviewer: OK, any other addition? Tell me about the frequency of meeting with institutions (district assembly, social welfare department, health insurance)

Respondent 5: Some time ago health insurance came and renewed our cards for us. That's what I'm even using currently.

Interviewer: OK, any other addition

Respondent 2: We used to work with health insurance scheme and I'm still using their card too.

Interviewer: OK, any other addition?

Interviewer: If there is no other addition let's continue. I hope I have your attention.

Respondence: Yes, we are focused. You can continue.

Interviewer: OK, now to my next question. Please tell me about how you collaborate with governmental stakeholders in mental health care. **Probe** on the last time such a collaboration was held, where, and who participated.

Respondent 6: How we usually meet our doctor. Every day our doctor is always at his office, but the medication always varies. Sometimes he can give you 3 weeks of medication, sometimes 2 weeks or even a month's medication. So, every day the medication varies depending on your condition. So, he is always at his office anytime your medicine finishes, and you go you will meet him, so for me, the doctor is always available and is good for us.

Interviewer: OK, not only the doctors. How about the district assembly? How many times have they come for meetings; even social welfare how many times have they attended your meeting?

Respondent 2: Our doctor always gives us medicine. Gubkatimali also supports me.

Interviewer: OK, any addition? When did BasicNeeds come here? How many times has BasicNeeds come here? How many times have they supported you?

Respondent 3: Truly I used to go to the hospital for the drugs but now I don't pick drugs there. Sometimes I will let my brother send me to Tamale so that I can get an injection there. But now Truly I'm fine because the way it used to be, now is not like that again.

Interviewer: OK, why don't you go for drugs in the hospital again?

Respondent 3: I used to go for the drugs, but they told me to go to Gurugu and get an injection so now I usually go to Gurugu for the injection.

Interviewer: OK, let's talk about district assembly. Have you gone to the district assembly before? What was the discussion?

Respondent 2: I want to still talk about BasicNeeds because they are those who always come here. We are always in touch with BasicNeeds all the time.

Interviewer: OK, when was the last time they came, and what was the discussion about

Respondent 2: Ok that was last year. They gathered us and taught us how to handle and live with a mentally challenged person. They trained both the caregivers and the patients**.**

Respondent 5: Yes, they came, any time we hear of a meeting is BasicNeeds, no other organization has called us for a meeting, they and Gubkatimali always gather us, and any time they gather us they train us and encourage us to always come for meetings.

Interviewer: OK, to my next question. Tell me about the skill training they gave you to help you financially.

Respondent 6: The skill training we had was, first they gave us money and we bought life stock out of the money and used some to farm, they later came and inspected my harvest in my house, and they took some pictures of the farm produce. I still have evidence of their visit.

Respondent 5: What I witnessed was they told us to try and keep our privilege fund active. What I have experienced in this condition is, I went and sat somewhere and was just talking loosely and someone said look at how I went and mixed up with some people. It didn't make me happy.

Interviewer: OK tell us about the skill they have ever trained you on

Respondent 2: Gupcatimali they came and gave me Gh₵600 to be used for farming and is about 4 years now even Mr. Adam from (Basic Needs Ghana) came and took a picture of it.

Interviewer: Calling for more responses

Respondent 1: They gave me 400 cedis, and I used it to buy two goats for rearing as I speak, they have increased in numbers, and it also offers support to the group.

Respondent 4: They gave us Gh400, and we bought 4 goats, Unfortunately, they stole some leaving only one.

Respondent 3: They also gave us GH400 each, then my husband took it and bought two sheep. One died and the other is missing.

Interviewer: The support is many indeed. Now to my next question. What skill training do you want in this group?

Respondent 5: We want money to do a trade because we sell a lot of stuff, Also A Sickler cannot save money because the sickness will consume all the money so because we are in to trade the money is not stable, so we need a little push.

Respondent 2: What we need from you people is I am a farmer and also rear animals so I will need money support so that I can farm well and also take care of the animals.

Respondent 1: Yes, I am skilled already. I repair ceiling fans but due to the lack of money, if you want to venture into a different business, you can't.

Respondent 6: For me the skill I think that would perfectly fit as is as you know for a Sickler all the time you have to be washing, so I'm begging if they can help us and teach us how to make soap. For the soap making I think everyone in this group would benefit from it. if you know how to make soap, you can make and sell and also use it yourself. So, I think soap making will be best for us.

Interviewer: Calling for more responses

Respondent 5: Soap is our main issue because when sickness triggers, we usually fall, and our clothes get dirty. So why don't we know how to make soap, we can use it ourselves and sell it.

Interviewer: Let’s move forward and leave the soap.

Interviewer: How do you involve the community in the operations of SHGs activities? **Probe** for who in the community is engaged, how are they consulted/involved, when are they engaged, what roles the community members play, etc.

Respondent 6: How we are involved in meetings with our community, or the town is sometimes when there is a meeting and we are part of the meeting, they do announcements in the mosques, churches, and radio stations (simili radio). They usually do that and tell us about the agenda of the meeting and what we are also supposed to do sometimes the agenda can be on sanitation and when we join, we do go around and keep our community clean.

Interviewer: Any more contributions?

Respondent 2: This is how we get into our society, The radio stations help, and also, we those in the villages through the gongon beater. The gongon beater will give us that information about the meeting. And this is how we get involved.

Interviewer: Thanks, and let’s continue. Please pay attention to the discussion.

Interviewer: Please tell me about any health campaign activity that you involved the community. **Probe** for names of health campaign, when it was implemented, for how long it was implemented, what were the challenges, and what were the benefits.

Respondent 6: Yes, we have some in this community. Some time ago we had one durbar at the secondary school in this community, the country director was in the meeting, a representative of our chief was there, and all SHGs in this district were present.

Interviewer: When did you have this campaign?

Respondent 6: It has been a while. Many of us were allowed to advocate and let the people know that the condition was not transferable. So, if you are with someone and the scissures start, you don't have to run and leave him, you can help him to lie down for a few minutes it will be over. Unlike those who thought when the sick flush Nate and you are close by you will acquire the disease, they were sensitized it that is not so.

Interviewer: What were some challenges organizing it?

Respondent 6: There were many challenges since it was more about the sick It was difficult without funds even to the extent of having this dignitary, we had to source for help from imams and pastors, and the DCE Assembly members helped us with some canopies and chairs for us to have a successful campaign.

Interviewer: What are some of the things you think if we do the group will progress?

Respondent 2: What can make this group progress is money. Our main problem is money.

Interviewer: Money to do what specifically?

Respondent 2: To help the group.

Respondent 6: If they can give us skill training it will help in our well-being because they can't be giving us money all the time. But if we are working, we can get money through our work.

Respondent 4: We plead that you help us with the skill training. Training like tailoring, carpentry, Maison, or any other training that will benefit us.

Respondent 3: On the skill training this is my view, if is soap making, you will be able to do it very well but if it is any other skill some of us cannot. To me, I think is only the soap-making some of us can do.

Interviewer: What are the facilitators of SHGs activities?

Respondent 1: I think if they help us with the soap-making, it will help this SHG.

Interviewer: Is it only soap? Bring out more views!

Respondent 5: If you give us money, we will know what to use it for so that it can generate income for the SHG. You can follow up for evidence of the income that it would generate since you want proof. So we just need some morning so that we will know where to invest it in.

Interviewer: What are the barriers to SHGs activities?

Respondent 6: What will make this group dissolve is when we don't attend meetings, If we sit to discuss it will help the group to grow but if we meet always we will be able to discuss the way forward and also keep in touch with each other.

Respondent 1: That's true if we attend meetings this group will grow but if we don't this group will die off.

Respondent 2: Meeting attendance is very important because we are from different places and is the meeting that will help us get to know each other.

Respondent 3: Truly attendance at meetings will help us know each other better but if we don't meet always, we will not know the way forward.

Interviewer: What are your challenges?

Respondent 5: When we meet, also try to visit us regularly, it will help encourage us and also encourage you as you know we are doing our best. Anytime you call us we will come.

Respondent 6: What I want to add is everything in this group is centered on taking drugs. These days getting the drug it's difficult and if you don't get the drug, we will not be able to come to meetings because we won't be in good shape.

Interviewer: OK let's continue. Please hold her so she doesn't fall (one had the attack). Please pay attention.

Interviewer: What role do you expect government agencies to play in meeting the health and social needs of people with mental health conditions?

Respondent 6: What we want the government to do is, we don't have a mental Health facility around, so we appeal to the government to help us.

Respondent 2: The hospital is our main problem.

Interviewer: What do want the government to help you with?

Respondent 4: I will say that drugs there are days that you go to the hospital and the drugs are not there, you have to go and buy them and if you don't have money to buy that would be a problem. So, the government should help make that drug available in the hospital.

Interviewer: We are edging closer to the end of this interview now to my last question. What are your SHGs doing to ensure sustainability?

Respondent 6: The skill training we talked about is what will help us to sustain the group. Is the skill that will help us get small money to support the group.

Interviewer: Any additions? What do you think you can do to sustain the group if you no longer get support from the government?

Respondent 4: We are appealing to the government to help us with the skill so that when he no longer supports us, we can support ourselves to get that drugs.

Respondent 1: Actually, some have the skill but others don't so I think those who don't have if they are also trained it will help this SHG.

Respondent 2: The skill training is a good suggestion so if many are skilled our groups will not dissolve.

Interviewer: What are other recommendations for effective SHGs operations?

Respondent 6: Like we said others are hidden so if the government gives us a mental health facility it will help bring many out to take their drugs and it will show that we are passionate about each other.

Interviewer: Any other additions

Respondent 5; Like my brother said to grow you would have to help us in business. As we attend our meetings, we may meet someone and the person will ask when our next meeting is again, so Because we meet it helps us create that scene of relationship with each other, so if there is no support and we are not able to meet then the group will die off.

Interviewer: I want to thank everyone for the time and for sharing your ideas with us. First of all, I want to thank you, and may the lord bless you, I want to stand on behalf of the researchers to thank you. May God help you and grant you a speedy recovery.

Respondent: I also stand on behalf of this group to say thank you to basic needs for always keeping us and supporting us God bless and keep you. When we were home with the sickness, we didn't know what to do but when you came and showed us how to handle it, we saw changes God richly bless us all. Amen.

FOCUS GROUP DISCUSSION

Interviewers: Good afternoon to you all. My name is ……. I came with my colleagues who are, my name is …... I'm ……. BasicNeeds Ghana has sent ……. to come and take this information from you and he has also sent us to stand on his behalf to take this information. This afternoon we have a few questions for you. It will help us know what goes on in this group. When we start this group questioning, we'll give you numbers. When you are to respond to a question, mention your number and respond to the question. When I ask a question, any of you can raise your hand, mention your number, and give your response. I will start from my right side, my grandfather you would be number 1, you would be number 2, you would be number 3, you would be number 4, then number 5 and number 6. When you get up to answer a question don't mention your name mention your number. Our first question is.

Interviewers: How do your SHGs operate within your community (ies)? · **Probe** for times/days of operation, membership, convening of meetings, frequency, participation during meetings, how resources are sourced, etc.

Respondent 1: I'm from the………community and I'm part of it. The meetings we have are always scheduled every first Friday of every month.

Interviewer: Any other opinions?

Respondent 2: We are ……. group and we are part of the group because of our health, and we are there because they always help us with drugs and the time, we will take their drugs and get well. Today we are working.

Interviewer: We would like you to tell us the time you have your meetings or days and when you sit for the meeting, what you always talk about, when you sit for the meeting what's your total number so if anyone can answer you can add your answer. Yes, my mother, you can answer. OK if you don't have the answer when they talk keep quiet.

Respondent 3: In this group, our meetings are always on the first Friday of every month.

Interviewer: So, when you sit for the meeting what is always your number?

Respondent 3: Our number is 20.

Interviewer: We are moving forward.

Interviewer: Describe your typical day as someone with a mental health condition or disability too. When you wake up how does your day look like? What time do you go to work, what time do you come from work and when you go to work what do you do?

Respondent 3: We are farmers We don't go to the farm in the afternoon we go in the morning by the time the sun gets hot we will be back home because when the sun is scorchy it can trigger our sickness.

Interviewer: Good, any other addiction?

Respondent 4: As a person living with a mental health disability when I can wake up and I'm not feeling very well I have to sit down till I'm calm before I can get up. When I get up, I go home straight I will not let the sun scorch me.

Interviewer: Any other additions?

Respondent 5: Because I'm not well when I go to the farm and I'm farming my hand would be shaking, when that happened, I must sit down slowly and wait till I'm OK sometimes I have a child with me so when I'm struck with the sickness, she ties me down till I'm OK. immediately after I recovered, I would tell her Let's go home and we will go home.

Interviewer: What are some of the unpleasant experiences you have had as someone with a mental health condition or disability?

Respondent 3: My sickness doesn't like a lot of noise so when I see people gathered around and they are making a lot of noise if I go there, it will start

Interviewer: Any other.

Respondent 1: The disheartening thing about this sickness is that you can wake up and the sickness will start. when I'm not able to get money to buy drugs or when I lack money to get drugs it causes me a lot of distress because the sickness will start and that is the unpleasant thing about living with this condition. Most of the time it's caused by poverty.

Respondent 2: With my sickness sometimes fluid comes from my nose when we are gathered like this the fluid will choke me but I can't flush it out because of a lot of people I have to walk out to do it and I can't also swallow it back to my stomach I have to grow it out additionally when there is crowd somebody can just walk past you and you in here they are perfumed into your stomach it can trigger a sickness also, in my workplace when the sun is scorching I have to sit at a place of shelter or when I'm working with people and I realize this is going to trigger my sickness I will disassociate myself because if I force myself to work till the end of the work the sickness will be visible for everybody to see

Interviewer: Good, we are grateful to you all for opening. We are moving forward.

Interviewer: What are some of the pleasant experiences you have had as someone with a mental health condition or disability?

Respondent 2: What makes me happy is when I wake up in the morning and is still cold, I feel so good (laughing) but when the sun rises, and you say Let me get up and do something small to get money than your body will be weak and it gives the sickness more power

Respondent 3: Because we are farmers we must get up and go to the farm early because when the sun rises and becomes scorching it triggers sickness.

Respondent 1: About this sickness what makes me happy is that in the morning I have to check myself to see if I'm fit, if I'm done checking myself by 6:00 AM to 8:00 AM I will know my health status and know whether I can start work but if I realize I'm not in good shape I will leave the work

to God and pray to Him (God) because He takes care of His children, I will not stress myself and add more troubles to my troubles. That's how I observe my day when day breaks.

Respondent 6: My sickness starts when I'm sleeping or I'm about to sleep It can wake me up from sleep and sometimes when I'm up I'll be talking to myself, when that happens and I am able to sleep back I will be OK Additionally Thursday, Friday are bad days for me.

Interviewer: I'm happy with the way you answer the questions. We will move forward.

Interviewer: Please tell me about the operation of SHGs

· Probe sources of funds, funding stakeholders, frequency of receiving support, last time support of received and by whom.

Respondent 2: Where I get my money to do my work is in our group because they have taught us that we should work small, small. so that we can get something small to eat, when I go to get the drugs every day, I take one in the evening when that drug is finished and I'm not able to get it the sickness comes back.

Interviewer: Please if anybody gets the question very well you can answer. It seems like we are deviating.

Respondent 1: What gives the groups money is when we agree in unity with those who are coming to support us. the support is not always much but we have faith and when you have faith and you are in this group we can contribute one cedi each, Some time ago we instituted that but it didn't work. The contribution will help sustain us when the people supporting us are not able to support us again.

Interviewer: OK I'm not cutting you short, but do you get support from NGOs?

Respondent 1: OK we get support from Basic Needs.

Interviewer: Which period did they support you?

Respondent 1: The last time they supported us was four years ago before COVID-19 came and broke it. Sometimes they used to send someone from Accra to come and be in our meetings, but COVID-19 broke it and that was four years ago.

Interviewer: OK we are grateful

Respondent 3: Before we got any support, we used to contribute 1 cedi each. so that it can help when the support is delayed but now, we can't afford the 1 cedi again.

Interviewer: Good, let's continue

Interviewer: Now I want us to talk about our Collaborations with government institutions (district/municipal assembly, social welfare department, and national health insurance scheme)

Please tell me about how you collaborate with governmental stakeholders in mental health care. Do you frequently meet with the district assembly, social welfare department, and health insurance?

Respondent 1: Yes, we collaborate with district assembly, social welfare, and National Health insurance because there are times our health insurance will expire, and health insurance would let us gather all the cards and they will help renew it for us at no cost and this has happened twice and I'm a witness. Also, with social welfare, I witnessed one person in this group who was helped.

Interviewer: Which period did social welfare help him?

Respondent 1: that was four years ago, how that person got help from social welfare was when we heard there was a new common fund, and we heard people with disabilities have received help. some received grinding meal machines and got money to put up a structure for them, even to the point that some people went and attacked social welfare to help them get a tractor, They are a group at …… and social welfare refused to adhere to their cry. We had a general meeting at …….. and that was where we had this information. One person among us got that support from the district assembly and that was when they were giving freezers, sewing machines, and cattle to persons living with disabilities. Only one person among us got a fridge from the district assembly and by then our number was around 25. It was during this meeting we heard that persons with mental health conditions also received support from the district assembly and there was tension. When we heard that we planned that we would go and demonstrate at the district assembly that coming Monday, I knew the information would get to the director. So, when it was Monday, we picked our leaders to meet up with the director and ask him. It was during this meeting that one of us in this group received something from social welfare and not the district assembly. Also, it was once one of our members visited the district assembly and met them sharing freezers and he also got some by replacing someone who wasn't there, later Some of the group members came to ask me about it mostly nothing happens in this group without my knowledge so when they came to ask me about the fridge I told them I have no idea and when I later investigated I realized that he was also lucky, he went and met it at the district assembly so I explained to them that he was lucky so we should let it go. Social welfare didn’t relax there, they looked for the person whose name was supposed to receive the fridge and give him one and resolve the matter. These are the two things I've witnessed from the social welfare department.

Interviewer: Tell me the last time such collaboration was held, where, and who participated

Respondent 1: When you are talking about groups that have come to train us, basic needs have come to train us before. They came and taught us ways to get hand skills so that we would not be dependent all the time but they don't always come and pick some individuals out to learn soap making or making pomade, they don't always come and select some individuals from the group but they always support some of us with sewing machines and that is part of the skill training and you can’t ignore it. BasicNeeds have done these things for us.

Interviewer: Good, let's continue to the next question

Interviewer: What type of training do you think this group needs so that it will help sustain its members or people living with mental health conditions?

Respondent 1: I will answer this question too.

Interviewer: What type of training do you think this group needs so that it will help sustain its members or people living with mental health conditions? So that it can give you money so that anytime you need money to buy something you can get your own money

Respondent 3: In this group if we get Support from a different source, we would have looked for support for ourselves.

Interviewer: What type of skills do you want?

Respondent 1: The skills that we need are. When we get support with money, we can look for the type of skills we want

Interviewer: clarify the question. What type of skills do you want from basic needs?

Respondent 1: There are different approaches to getting skills. When you support someone with money that's the skill you have given the person because when you bring the money you will not say we should take it and you just go you let us know what we are going to use the money for and it is acquiring new skill you can also let us know that when we are spending our money let's not forget about the bank because sometimes it's in the bank that will save our money

Interviewer: I'm not limiting you but this is how we want you to answer the question. We would like you to clarify what exactly you want basic needs to train you on for example should basic needs train you on hairdressing or carpentry or weaving? that's what we need because if you are a hairdresser you can go and get money from that place so tell me what type of skills do you want for this group?

Respondent 1: This thing has worried us for a while now because it is, tailoring and carpentry. Some of our members are already in that business and they don't make any money, For example, imagine we all having tailors in our house who will give their clothes to others to sew so and Besides in this community when we have sewing machines they don't want to give us their clothes to sew because they believe we are mentally not sound some will not even give you money because they believe you are mentally not sound you don't know how to count money so it's not our will that we don't want the tailoring training but if we collect it, we can't use it to make money so it will not serve its purpose.

Interviewer: We have taken notice of your answer, any other addition?

Respondent 2: we need support. Support towards clothes weaving I want that type of skill additionally I'm a bicycle repair That's where I get something small from but when the sun gets scorching, I'm not able to work so usually I work under shelter.

Respondent 5: If the support is available for us, I don't stand in the sun for a long because when I’m hungry it triggers the sickness so I would say if I could get some of the lady's cooking stuff I could be doing that business while I'm inside the house so that anybody who needs to buy something can just walk in and buy. So, if I get money I will do business because if I step outside the sun it triggers my sickness.

Interviewer: Good I like the way you are answering the questions now to our next question

Interviewer: How do you involve the community in the operations of SHGs activities?

Probe for who in the community is engaged, how are they consulted/involved, when are they engaged, what roles the community members play, etc.

Respondent 1: With this question we have those we add to our lives just to promote unity some time ago they gave us a garden and they told us that when people who are not part of us want to help us we should allow them to help us so we didn't deny them we announced for them to come and help us in the garden and some people came out to help

Interviewer: Great and there are other additions?

Respondent 6: Since I got this sickness when I'm in with my peers they don't stigmatize me. My sickness doesn’t fear crowds, my challenge is just when I'm about to sleep. When it comes to a gathering where we are going to cook, I joined them to do so. in the crowd, I can sit and relate with everybody, and nothing will happen to me but when it's time to sleep that's where the problem is. with the way my sickness reacts if I get at a sewing machine or trade or selling of bread (that can you give me money too) with the rest I can’t go out of the sun

Interviewer: Great, let’s continue

Interviewer: Please tell me about any health campaign activity that you involved your community.

Probe for names of the health campaign, when it was implemented, for how long it was implemented, what were the challenges, and what were the benefits.

Respondent 3: Yes, they gathered us to teach us that when someone this struck with the sickness, we should use cloth and tie the person down so that the head will not hit the ground because when the head hits the ground it can increase or give the person a different sickness.

Interviewer: OK any other

Respondent 1: Yes, Doctors have come here to encourage us and show us how to handle our sickness so that people will not stigmatize us in the community. They usually let us know how to keep ourselves. When your brother falls on the way and you ignore him and leave. it means you have stigmatized the person already. They made us know that the sickness does not transfer from one person to another. This was what the doctor’s research showed them. the sickness is not transferable. So, when someone has this sickness because the doctors have educated us about it we have faith now. Ladies that are living with this sickness couldn't get married but because the doctors have educated us on it now, they are giving birth. The doctors showed us how to live with it and it does help us.

Interviewer: What were some of the unpleasant experiences you have trying to access health care as well as social protection avenues or opportunities? **Probe** for specific situations, when it happened, who was involved, etc.

Respondent 3: Someone called me a mad person and it hurts me I couldn't eat that day even though there was food, and it happened just in our community.

Respondent 2: there was a gathering, and I also went to the gathering it was in the hospital at …….. and they told me to go to the hospital, I went, and they checked me and gave me the pattern I should take my drugs, they said this drug should be taken ones every evening and they encourage me to keep taking it till am well. They picked someone in the hospital to write our names down, anytime it was time to take the drugs the person would go around and wake us up to give us the drugs, that was how it was till we left that place. When I go to the big hospital because there are a lot of people there who have come to seek medical attention when I inhale their perfume, it triggers my sickness, so they separate us in the room. Since then, I have not experienced the sickness again.

Interviewer: Good has anyone also experienced the same?

Respondent 4: When my sickness is about to manifest and I'm on the way it will roll me off the way and I'll fall.

Respondent 1: (clarifies her by asking) If it happened at the hospital.

Respondent 4: I went to the hospital, and they gave me drugs to take, after taking the drugs I was sweating and later my body was calm, and we went home. When the sickness is about to manifest it will start by making my heartbeat very fast then it will put me to the ground. Now they have identified the sickness, and they give me drugs to cure it.

Interviewer: Good Any other

Respondent 1: My sickness is this way, in the hospital at times the sickness disturbs me till I visit the hospital. They admitted me when I got there and kept me on drips till, I urinated on the bed. The sickness is serious, but the doctors play with it. So that happened I wasn't happy.

Interviewer: Good, let's proceed. What are the facilitators of SHGs activities?

Respondent 1: If I'm going to respond to this question. What we are going to be doing in this group that will make us move forward is peace and trust concerning work and hard work. If we put these first the group will be strong and move forward

Respondent 3: In the group if we can sit and consult each other the group will be able to move forward.

Respondent 2: I'm just there when am not feeling well and someone told me that if you don't plant trees, you will be struggling that's when I planted a cashew tree and added mongo trees too. Today when is dry season I make up 3 -4 bags of cashews and can sustain me for a while.

Respondent 3: When we get another NGO to come to our aid, it will help and make life easier for us.

Interviewer: Good, we are moving forward. What are the barriers to SHGs activities?

Respondent 3: What prevents this group from moving forward is lack of work and hardship so because of that it does not make work move forward.

Respondent 6: What will make the group move forward is unity. When there is unity, we will go high. The lack of unity will not make the group move forward. Hardship too will not let the group move forward. If we are united and there is hardship, we can't move forward.

Interviewer: OK any other or we should continue.

Respondent 4: What will make the group go forward is when you help, we the poor. Some of our members don't have anything doing and we want you people to encourage and support(funds) us to come for meetings. This would help our sickness reduce, so we can move forward.

Interviewer: What can be done to address the challenges people with mental health conditions face in this community?

Respondent 3: To me, if could get an NGO that would come and help us with something, it would change a lot.

Respondent 1: With this sickness and struggle, we are crying to you (basic needs) people that Bugiya has a small hospital if the government observes and sees that we have a doctor in this small hospital. When the sickness is about to disturb the doctor can come to our aid. If the government can help us with that it will reduce our struggle. The clinic would help us because everyone is crying there is no money. Where there is sickness and struggle, money is needed.

Interviewer: What role do you expect government agencies ***to*** play in meeting the health and social needs of people with mental health conditions?

Respondent 3: To me if He could get us a hospital it would help in emergency cases either evening or morning.

Respondent 2: If He can get us the drugs, we have a hospital where we can keep it there so that if anyone needs it you can get it there. There are times you would go to buy the drugs, and you won't get some and when you come home without it(drug) you can't sleep.

Interviewer: Good is someone having a different opinion?

Respondent 3: Transportation to the hospital is a challenge for some of us. To get even a bicycle to go for drugs in the hospital is a challenge.

Interviewer: Good, let's move to our last question

Interviewer: What are your SHGs doing to ensure sustainability? What will you do without support from NGOs such as BasicNeeds Ghana?

Respondent 3: What we will do is, if the group can still maintain meetings and be able to discuss and contribute something(money) down.

Respondent 1: Looking at BasicNeeds, if they are going to take off their support. Even duo we are sick we have students among us. If they can help our student, some of our students are graduates (JHS, SHS) and needs support to continue. If they can help us with that and now leave the students they helped can now take care of us.

Interviewer: Good is someone having a different opinion?

Respondent 2: If they don't help us again, our garden, they gave us some skills about it. We have knowledge in that sector that is helping us get something to feed on.

Interviewer: So that's what you would depend on for survival?

Respondent 2: Yes, if we agree and continue with the garden and there is fertilizer or fertilized land. We can continue with the garden.

Interviewer: Any other recommendation for effective SHGs operations?

Respondent: We want animals to rear

Respondent: If we get Goats, Fowls, Sheep, etc. to rear. It can help sustain us.

Interviewer: Mommy it seems you have something to say

Respondent 4: If you can do that when the sickness strikes, we can use the animals to make some money for our treatment.

Respondent 1: What I will encourage is some of us are traders so if you give them animals to rear it will be hard for them since they don't sit in the house, so I will say they should give us money to do trade or better still they should give traders money and give those who want animals to rear animals. They should help everyone according to their need.

Interviewer: I stand on behalf of researchers to say thank you for your time and may God bless this group. God bless you all.

FOCUS GROUP DISCUSSION

Interviewer: My name is ……. and these are my colleagues, ……………. We work for Dr. ……. Today we would like to have a conversation with you all. We are very interested in learning about the operations of SHGs for people with mental health conditions and disability. We will only share the information we learn today in a general way that does not reveal the identity of anyone in the group. (As a result, respondents were giving numbers to conceal identity). With your permission, I will be recording our conversation. It's important that the information shared in the group does not leave this group. So, we ask everyone not to share who was here or what was said the group with others outside the group when you leave here. We really want to hear what you have to say and want you to feel comfortable in answering questions however you want to. There are no right or wrong answers.

Wumpini would be taking note to make sure that we don't miss what you have to say. This will help us later when we go back and organize all the information that was shared today. The group discussion should last for 30minutes.

Interviewer: How do your SHGs operate in this community (ies) operate?

Respondent 1: Truly, our group is very supportive of one another, the help is that when one has an occasion the group goes to know how the occasion is. Occasions such as someone has a naming ceremony, or funeral.

Interviewer: Great, we would want to know days/times of meeting,

Respondent 1: We meet the first Saturday of every month.

Interviewer: How many would your membership be?

Respondent 1: Both the sick and care givers put together; we are over 60 people.

Interviewer: How are resources sourced?

Respondent 1: In terms of resources, BasicNeeds Ghana gave us chairs and canopies so when we have a meeting we use it. But in case someone (none member) has a naming ceremony or wedding and needs our chairs or canopy, we rent it out to them and make some money for our group.

Interviewer: Anyone with something to add?

Respondent 5: What he said is true, this is number 5 speaking. When one of us has a need of it and comes to request for it, we give it out. But if it someone comes to hire it, the money paid is kept so when one among us has a challenge we use that money to support that person.

Interviewer: Do your members attend meetings very well?

Respondent 5: By God's grace and power, they come.

Respondent 4: Truly, our group is very good. Anytime it's first Saturday, everyone usually come out in their numbers for meeting. True chairs were donated to us and I have even used them for a naming ceremony.

Interviewer: Which people gave you those chairs?

Respondent 4: BasicNeeds Ghana, I find it difficult to pronounce the name (laughs). When I had a naming ceremony, I was supported with the chairs because I didn't pay for it. So our group is so helpful to us.

Interviewer: Describe your day as someone with mental health condition or disability?

Respondent 2: Truly, as for me, this is number 2 speaking. When day breaks, the only thing I do is lying down. When it is time for salat, I go to pray and come back to lie down again. I don't have any business. To me, I have no business to do.

Respondent 3: When day breaks, I have a small business I operate. I sell provisions. After dawn prayer, I come to open the store to sell a little and I close around 11am.

Respondent 6: This is number 6 speaking. I'm a welder. I work from home. The little I earn is what I use to take care of myself, my health and everything that concerns my life. I leave for work by 7:30am. Everything is going well at my work site. I close from work by 6pm. This is what I must add concerning my work and how my day goes. May God bless us.

Respondent 1: I am a bicycle fitter. Some mornings, there could be an occasion such as naming ceremonies, or weddings at home, as a result, I may not be able to leave home early, sometimes I leave home around 9am and get back around 6pm that's how my day goes. Sometimes I can leave the house around 7am thus I don't really have a specific time I leave home for work. When the day breaks, sometimes issues at home can delay the time I want to leave for work, so I really don't have a specific time I leave for work.

Interviewer: What are some of the pleasant experiences you have had as someone with a mental health condition or disability?

Respondent 3: BasicNeeds came to support me I was very happy. I have six children and BasicNeeds came to support me with five hundred Ghana cedis so it could help to be getting some porridge for my children. That day I was very happy because I didn't know what to do and they came to give me such an amount of money, I was so happy.

Respondent 5: Truly, what BasicNeeds have done for us we wouldn't be able to say all except God. They gave me support and that support has kept me till date and I thank them so much. They called me and supported me with two thousand Ghanaian cedis that I should take the money take care of my daughter to learn tailoring and the education of the other siblings. I went for the support and I'm thanking and praising BasicNeeds Ghana so much.

Interviewer: Anyone with something to add?

Respondent 4: What they are saying, I have only heard but I have not seen it. I haven't had any support. I wouldn't lie. As I have not had support, what is it, it is because when we meet, I only hear of it but I have not seen it. Even in Sagnarigu we met.

Interviewer: Not only BasicNeeds Ghana, haven't you had any pleasant experience in this community?

Respondent 4: oh ok, I have had, I have had

Interviewer: And what is that experience?

Respondent 4: My friend did something to me, and I was so happy.

Interviewer: what did he do for you?

Respondent 4: She gave me food. She gave me four bowls of maize. That I should use to feed my children because I don't have much to give them. When it finished, I went back, and she added me more maize and I came back to take care of the children as their father is no more alive.

Interviewer: Anyone with something to add?

Respondent 2: Mr. Sandoo and co came to take me out of ignorance into enlightenment. What I mean is that how to eat was a challenge, what to wear was another challenge, going into the public was also another challenge. Why I say this is that they came to assist us with animals. So, the animals, I was rearing them. Because I have been falling it affected my brain, I went to the hospital, and they said that my head has been injured and so they were going to give me medication. And the drugs, each tablet would cost me ten Ghanaian cedis. I was rearing the goats, and they were there. My father, the only thing he does is farming so he doesn't really care about me unless I am the one who talks to him that I am sick. So, when it comes to buying the drugs, I usually talk to chairman telling him how my condition is thus I want to pick one goat to sell and go to hospital and then he would say, "Organiser you can pick. The support they gave you is for your health so pick one of the goats to go take care of your health." So, to me they took me from darkness into light.

Interviewer: Moving forward, what are some of the unpleasant experiences you have had as someone with a mental health condition or disability?

Respondent 4: Someone can just see you and stigmatise you. Are you getting me? Someone would just see you and stigmatise you or sometimes when you eat food and there is left over, they would ask you to pour it away. That you should pour it away. Or someone would meet you on the road and look at you in a very demeaning way because of sickness meanwhile you are older than such a person. May God help you to help us treat our sickness.

Respondent 2: As number 4 said, truly when I sat among my friends they called me mad person. This number 2 speaking. Whenever I sat with my friends they called me a mad person because I don't talk. I always sit quietly among them. I met Mr. Sandoo and co. When I was to join the meeting, the women leader took me to chairman and chairman and the women leader took me to the hospital. It was there I got to know about the meeting and began going to take medications. Each time I went, they would give me medication for a month, other times two months, other times four months, and I was taking the medications. As I take the drugs, one day I went to the hospital, and they told me that I should pay ten Ghanaian cedis. They said the drugs is no longer free, that when you come to take the drugs for free, you must pay ten cedis for them to write a note for you so that you would be able to come for the drugs for free. So, I told them that if I had ten cedis I wouldn't have even come to them. The one who is head of the unit at the hospital began shouting at me that what I had said is nonsense.

Interviewer: That was your unpleasant experience?

Respondent 2: Yes, I was very displeased and went home.

Interviewer: Anyone with something to add?

Respondent 6: Truly speaking, I have seen life and how everything is going and wish to bring this matter before my father because my father didn't know about it. There were times I wanted to go to my workplace, it was at work place they saw how I lived and told me that for me, I'm such a weak person and that they don't know what is wrong with me. I told them what was happening to me in my life, and they didn't believe it. There were times we were to work, they would be looking at me a way saying, "So-and-so why are you like that?" So, I informed them about my condition then I came home to inform my father. My father took me to the hospital. I began taking the medications and saw changes in my condition. That was when I realized how the drugs can bring a lot of relieve. That is what I have to say.

Respondent 3: Our sickness has a lot of stigmatizations. Even in my home, I have never been stigmatised. Sometimes when I cooked food, no one would eat the food because of my sickness. Even at times water in my pot, people didn't want to drink it. They think they can get sick if they drink the water. Because of this, I went back to my father's house. Now I live in my father's house because of the sickness and its attendant stigmatization.

Respondent 5: Truly, the sickness has a lot of stigmatizations to it. Even I number 5, in my house when day breaks, my children with me are two. It is only the children and I who converse in the house. My siblings and their wives don't converse with me not because of anything but sickness. For the sickness I thank God a lot concerning the way they stigmatise me because of the sickness. Even in the mosques people don't want to stand by you. In my house when there is an issue, it outside I would later hear it and no one would inform me because of the sickness.

Interviewer: That is what hasn't pleased you?

Respondent 5: That is what has displeased me. As a human being, you know your beginning, but you don't know your end.

Interviewer: We would like to know your source of funding, funding stakeholders, frequency of receiving support and the last time support was received and by whom?

Respondent 3: The last support we received was from BasicNeeds Ghana. They called us together just as we've gathered for this meeting and gave some cash support. They have also been coming out to support us with chairs and canopies and we rent them out. I am the treasurer. We rented out the chairs and canopies. They are those who have been giving us support. Anytime support is to come to us, it's mostly from BasicNeeds Ghana.

Interviewer: Does anyone have something to add?

Respondent 1: Where we get our funding from are many yet not many because we still need more support. BasicNeeds Ghana has been supporting us, Social Welfare, and the Metro Assembly. Last year, they supported some of us with cash, some had thousand Ghanaian cedis, others had thousand

five hundred cedis. In our group, they supported up to eight people. Those I know and can point out. It was BasicNeeds Ghana who collaborated and linked the group to social welfare and the assembly for that support to have been given. This is what I must concerning our source of funding.

Respondent 4: This is number 4 speaking, last year, they showed us kindness. When I say they showed us kindness, you know I'm an old person…

Interviewer: Which people?

Respondent 4: What is their name again, that is the assembly people. They called us and we wrote applications to them and the support came and we went. They gave us something small. As they gave us a small support, I'm an elderly person and not tough. If I keep the money, people will come and borrow the money and at the end, the money would be wasted. So, I gave out my money to be used to buy two animals. They were sheep. I took care of them and the multiplied to four. One day they ate grass sprayed with poison and two died and left two. Recently, one of the sheep went and chewed rice and died. Now it's left with one. It also got pregnant, and the baby died. Whatever the support is, it is also good. I thank God, when the one left populates, I can use it to take care of my health. If you have no money today and you are sick, you will amount to nothing.

Interviewer: What I want to clarify before we move on is from whom did you receive your last support?

Respondent 3: Our last support came from Metro Assembly. It was the assembly who recently gave us support.

Interviewer: You can't remember the year and month?

Respondent 3: It should be up to a year and half. Metro Assembly called us, I got thousand five hundred Ghanaian cedis.

Interviewer: very good. In number 1's submission, he mentioned something related to our next question. Yet I would want you to throw more light on it. I would want to know your collaborations with government institutions (district/municipal assembly, social welfare department and national health insurance scheme)

Respondent 1: We, BasicNeeds Ghana, social welfare, and metro assembly have met. We met for a discussion and came to some agreements. If I'm not mistaken it should be up to a year now. When we had our last meeting, the agreement was what the metro assembly would do to ensure they are aware of us so that anytime we come to them for support, they would be able to support us well and with enthusiasm. In that meeting, DCE was there, the head of social welfare was there. The name I can remember, Madam Hawa was there. Also, there was also a police representative, and I don't know which other NGOs, but they also had representative, and the meeting was held. We met at the metro assembly.

Respondent 3: When they called us for the meeting, this is number 3 speaking. When they called us for the meeting, a nurse from the Tamale Teaching Hospital (TTH) was there. DCE was also there. Madam Hawa was there. The Director of Health Insurance was there. The health insurance director told us that anytime our health insurance expires, we should put it together and take them

to Madam Hawa who oversees social welfare, and she would bring for them to renew it free for us. So when we met, they told us that thus anytime our health insurance expires, when you send it to the social welfare director, Madam Hawa, she would take it to the health insurance office and they would renew it for you. When we met them, they told us all this.

Interviewer: Please tell me about the kinds of training you have received to support you financially.

Respondent 5: Truly, some people have given training which would help us to support ourselves financially. Those who gave us that training was ……. and social welfare. They met us to talk to us. They were to give us animals to rear. They taught us how to take care of the animals so that we can benefit from the support. Because in these times, no one has anything in their rooms for the rainy day but if God allows and they give us the animals, and we keep them well and God helps us and the animals populate, the animals will be of immense benefit to us, so they gave us. When they gave, some people's animals died. Even I, they gave me two goats, but they all died. When our animals died, others were fortunate, and their animals did not die till today. They are still keeping the animals and it's helping them but some of us no longer have any animal.

Respondent 1: What I can remember is that BasicNeeds Ghana contracted NORSAC to train us in business. In the training, they told us that when you have capital to do business, you don't lend the money to anyone who just comes to borrow from you especially family members. Most of them won't pay and that can collapse your business. Others just come to borrow from you because they want to collapse your business. They also trained us around reproduction. If it's a woman, how to take care of themselves during menstruation. How to put on menstrual pad. All these, we were trained on it.

Interviewer: Describe to me the kinds of skills/competencies you will need to effectively support your operations as SHGs.

Respondent 4: As for the skills unless you come to train us.

Interviewer: Mention the skill you would need.

Respondent 4: As for me I process rice to sell. So, I would prefer more training in that regard. That is what I am selling. I sit in Aboabo market, Tamale, that is the area of competencies I would need. That's the end of my contribution.

Respondent 5: What she said is true, truly I'm not well but I grew processing and selling the rice. I sell at the market, but I have no finances it makes it difficult for the business to grow. So, if you had to give skills training in the rice processing it would have helped me a lot.

Interviewer: So, what the things you would for effective training?

Respondent 5: The thing we would need is money. That's our main problem, the money. It is not there.

Respondent 4: What she has said is true. If you don't have money, today, how much is a bag of unprocessed rice? How about a bowl? One bowl of unprocessed rice is twenty-five Ghanaian cedis.

If you are to buy ten bowls, that's two hundred fifty Ghanaian cedis. We need financial support so please help us.

Interviewer: Any other thing?

Respondent 3: The skill I would want you to support train us in is that you know some of us are now old and we didn't learn sewing, weaving, and the others. It is just the petty trade you have learnt. For the business, I sell provision stores and others for me and my children to survive on. But it needs money. If you could support us financially, we could use it to trade so some profit could be generated for us to survive on it with our children.

Respondent 1: You are saying the skills we think would be of utmost help to us for effective operations of SHGs. Among us, there are young men and young ladies. First, weaving, when you train us in that skill, it would help the SHGs. In the radio and television, if you find a way so we could come on air, we the sick and care givers to educate the public on this sickness. It would help a lot. When we talk about the fact that sickness is not transmittable, those who are usually afraid and when someone gets seizures around them, and they run away and leave the person. As everyone gets to know the sickness isn't transmittable, when someone gets seizures in public people would come around to assist them rather than run away. All these would be of immense help to us. That's what I must add.

Respondent 6: I would need training in farming or business or any skills training. This would help to make life go on well. With farming, every year would need support for the farming season. The support is money. When we get money, we would be able to farm, if we get farm implements or farm chemicals or any other knowledge, we would need in the farming sector. We would use all these in our farms. By god's grace, when we get a bumper harvest, it would be of great help to us and our families. This is what I have to add with regard to the skills training.

Interviewer: How do you involve the community in the operations of SHGs activities?

Respondent 2: This is number two speaking, the people we usually invite are Mr……. and co.

Interviewer: Here we want to know the people you involve and engage within your community.

Respondent 2: The person we usually invite is the assembly member.

Interviewer: What did you invite him for?

Respondent 2: We have invited him to come and teach us how to live within our community peacefully so that our lives would be better for us.

Interviewer: anyone with something to add?

Respondent 3: Our SHG has ever invited our Member of Parliament, Lawyer Haruna. He couldn't come himself, but he sent a representative to the meeting. We wanted him to come and know what we do because if you have a group and you are people who aren't well, it is good your leaders are made aware of you. As we are there, we've ever paid a courtesy call to the Chief of ……... So the Chief of …….knows about us. Lawyer Haruna sent a representative to visit to know about our SHG activities.

Respondent 1: What I am to add is that, in our community, how we involve them in our activities, as the community knows this is a group of people with mental health condition and disability, so anytime someone gets this sickness, they quickly run to us so we can help the person to access medical help. For instance, if someone gets epilepsy, because they are aware of us in the community, they quickly run to us for help. They ask us what they can do to access medical health either for themselves or relatives so they can get well. We provide such guidance. When there is a need to send the person to hospital, we do so. In our community that's how we are involved in the community.

Interviewer: Please tell me about any health campaign activity that you involved the community.

Respondent 5: Yes we've had a health campaign to talk to them. We told them how to keep themselves to prevent this type of condition. We also talked about the things they might do that can cause this condition which they don't know. For instance, there are some people, you know one cause of this condition is the way we beat our children. Some can be so heartless that they can beat a child to the extent of even hitting the child's head with a wall or any metal around. All these can cause this condition and some of us are victims of this.

Interviewer: So that was your message in the campaign?

Respondent 5: Yes, that was what we taught so that it would to protect them

Respondent 4: This number 4 speaking. May God have mercy on us. Do you see what happened, only God can lead us in the right path. No human being has the knowledge. There is no human being who can know things like this exist in this life. May the Lord have mercy on us and show us the way to cure our sickness. This sickness worries us a lot. If you have it, you can't go into the public domain. No matter how well you keep yourself, people will still stigmatise you. You go to stand with someone, they would be murmuring and asking why this person is epileptic and you're standing with them. Meanwhile, you didn't buy the sickness. It is the workings of God. May the Lord help and this sickness would leave our bodies.

Interviewer: What do you want to know about any health campaign you've had in your community.

Respondent 1: Last year, we gathered. We went to the ………Chief's Palace. The Chief sent out the "gongong" beater to summon the community and they gathered. BasicNeeds Ghana sent representatives. They call it durbar. They spoke to the people not to discriminate against us, they shouldn't stigmatise.

Interviewer: What were the challenges?

Respondent 3: When we were to have the campaign, BasicNeeds came and supported us because you can't go to a Chief's Palace without something to offer. They gave support so we could pay a courtesy call on the ……..Chief. We spoke with him to gather his people, and we talked to the people. That day, BasicNeeds even came with the drugs. We told them that this sickness is not transmittable so anyone who has it, they should do well so that there would not be discrimination and stigmatization. The ……..Chief also added his voice to talk to the people. We gathered the

people to educate them just as they have also been educating us. We take it home to also educate our community.

Interviewer: What were some unpleasant experiences you have trying to access health care as well as social protection avenues or opportunities?

Respondent 4: Truly speaking, as for me when I go to hospital, they don’t want to mingle with me. They know it isn’t transmittable. As for drugs, they give me drugs when I go. There are some of the hospitals they don’t treat us well (at the end her utterance she mentions the Tamale Central Hospital) but for the clinics, they are very patient with us, encourages us to always come back. They don’t usually deal with us in anger.

Respondent 6: I have ever been to a hospital and told them my condition. They gave me medication. I came home and took the drugs. It began to have effect on me. As number five said, you come home and get some unusual seizures and people look at you in a way you are not comfortable. When I go back to the hospital to tell them that the drug they gave me is having negative effect on me, they would change me a drug. They would change a drug and ask me to take that drug and there would be changes. I think that is what I also have to add.

Respondent 5: For me only one person ever insulted me in the hospital. I wasn’t happy with the insult. She was a nurse. I wasn’t happy so I drew her attention to it. I told her that what she has said to me is because of sickness she had the chance to say such things to me. For the sickness, she couldn’t have said such nonsense to me. So when they are doing their work, they should be mindful that there is a future. I went to take the drugs. The drug was there but she told me the drug was not there. The doctor had not yet come. I told her to write it for me. She retorted is it not a written note I already came with. I should use that note to go buy the drug. I said she shouldn’t have spoken to me like that. At the end of each month, when we come, they usually write it down, so they know so-and-so person has come. She shouted I shouldn’t disturb her. I said to her to remember there is tomorrow. Truly what she told me I wasn’t happy.

Respondent 3: We have a particular nurse. This is number 3 speaking. We have a particular nurse. Even our contacts are with her. In case she gives you the drug and you have negative side effects; you can call to inform her. The next time you go, she will change your medication. There was a time they gave me a particular drug; the drug was too strong for me. I called to inform her, and she said I should return the drug. I returned the drug, and she changed me a different drug. So the hospitals when you go, frankly speaking, when you meet someone who is sensible, they always encourage you and don’t want to say anything to offend you. This our sickness doesn’t want us to become unhappy. When they say something that can affect your mood, you can get seizures. They have to keep encouraging us till we get well.

Interviewer: Number 3’s response has taken us into our next question. What were some of the pleasant experiences you have had trying to access health care as well as social protection avenues or opportunities?

Respondent 2: Where a doctor spoke to me, and I was so happy was at the Tamale Teaching Hospital. I went because someone informed me a new doctor had come to look after people

suffering from epilepsy and mental disability. A friend gave me the information and I went to the hospital. When I entered, he bought Don Simon fruit drink for me. Gave me a sit to sit. He said I should make myself comfortable, finish the drink before I can tell him what is wrong with me. He said I should relax and eat the food. I ate the food. When I finished eating the food I was so happy with the treat. I told him I have been going to hospitals, but his reception and hospitality has made me so happy. He again took me to where I would take the drug. I felt like crying because he led me for everything to be done. And he even used his car to take me home. He added me money which I spent for up to a month.

Respondent 1: As for me, Dr. David Abdullai, may the Lord move his generations forward. In his clinic, this is number one speaking, I went, and they examined me. One day, I went there when I was to leave, he gave me honey. I enjoyed the honey so much. I was so happy.

Respondent 2: Let me add to Dr. David Abdullai's story. This is number two speaking. As for Dr. Abdullai, may God bless his generations. As for that man, I have not seen any doctor like him in Tamale.

Respondent 3: Dr. David Abdullai, when he was alive, received us and treated us for so long. Even food, every month when we went there, he would give us food stuff and add you the medications. It is his absence that has made us to go to the hospitals to buy the drugs. When he was alive, when you go there, he would give you the drug and add you food stuff.

Respondent 5: Truly, as for Dr. Abdullai, everyone is grateful to him and prays that God would bless his generations and raise others like him. Anytime you went to his clinic, you would take the drug for free, and he would add your food stuffs, and give you lorry fare or he would ask his driver to drop you home. He would tell you that when you take the drug, you should sleep for several hours and wake up at such a time. He would tell your family that they should take care of you. That they should be patient with you because it takes patience to take care of a patient. But shouting and barking at the person; the sick person wouldn't have peace of mind and therefore wouldn't leave normal.

Respondent 4: May God raise people like Dr. Abdullai. I used to take medications at his clinic and the distance became a challenge for me. He asked of the name of my house; my name and he said I shouldn't come for the medications again. He used to bring the drugs to my house. When he was to bring the drugs, he would mix with food. He would tell me to take two or one pill. I would take the drug. He would then advise me to lie down quietly because the medicine doesn't like aggression. When you take the drug and sleep, until 3pm in the evening before you wake up and your body would feel so fine. So may God have mercy on him. May God raise people like him.

Facilitators and Barriers

Interviewer: What are the facilitators of SHGs activities?

Respondent 1: To my opinion, if government had added we those with mental health condition and disability to the LEAP monthly allowance it would have helped us a lot to promote our activities.

Respondent 2: To me, as chairman has said, to my opinion, Mr…….. and team should make it a point every to send an officer to our meeting. It would have helped the meeting to go forward for us.

Respondent 3: To me, what would facilities the activities of the group is that as chairman already mentioned. If the government adds us to the LEAP monthly allowance it would help a lot to ensure that the group moves forward all the time and not retrogress. If members know something small will come at the end of the month, we meet on the first Saturday of every month so if we are receiving this allowance, no one will have to call the other to come for a meeting. All the time members would have been very active.

Respondent 5: What I must add is as chairman already mentioned. Government should add us to the LEAP monthly allowance. The majority of us have nothing doing. When day breaks, most of us have nothing to do.

Respondent 6: I thank my colleagues for their contributions. May God help and promote this group. I think this is just what I must add.

Respondent 4: You know how life is now. If you want to stand from the ground but don't support yourself with your hand, can you get up? The hand that can support us is the government. He should help us for God's sake because we don't know where to turn for help. Social welfare should help us. Metro Assembly should help us for God's sake. We don't have to eat. They should help us for God's sake.

Interviewer: What are the barriers to SHGs activities?

Respondent 2: They said we should be making some contributions. Last month we sat and agreed on the contribution, but I think it wouldn't help. Even what to eat sometimes is a challenge so how do we get money for such contributions. To me I think it is going to serve as a barrier to the progress of the group.

Respondent 1: What I would add is that when we call for a meeting and members don't come in their numbers it would not help the group to progress. Also, when complacency sets in either on the part of the leaders or the members, it can also affect the group and its activities.

Respondent 2: What I would add is that when there is no trust among members, it would be a barrier to the progress of the group. Aside mistrust, gossip.

Respondent 4: When you call for a meeting and everyone comes out. Whatever you plan everyone supports it. The group would progress. But when you call for a meeting and members don't come, the group wouldn't move forward. It would weaken. But the good news is that we have never called a meeting and members don't come in their numbers.

Respondent 5: What I think would be a barrier to our activities is if everyone wants to prove they are wise. In a group when everyone wants to show they are wise, if not a lot of prayers, such a group would surely collapse. The way we used to be as a group, we are no longer like that. When we say there is a meeting, everyone wants to prove they know it better. That way, it can collapse. I would want to plead with this group to stop such attitude.

Interviewer: What can be done to address the challenges people with mental health conditions and disability face in this community?

Respondent 5: For us those who aren't well in the community, to my opinion, what would help to mitigate our challenges is if we have businesses we are doing. It would have helped reduce the discrimination and stigmatization we face in the community. But when they see you doing nothing, they don't even add you to anything in the community.

Interviewer: What role do you expect government agencies to play in meeting the health and social needs of people with mental health conditions?

Respondent 3: What we want the government to support us with is to add us to the LEAP monthly allowance he gives to the poor so we can also benefit from it. When we are taking the allowance, it would help us a lot.

Respondent 1: What I would add is what government can do to help this our group a lot is that he has to make sure the drugs are available in every hospital and clinic so that any hospital or clinic we go to you would get the drugs for your condition. It would have helped the group a lot.

Interviewer: What is your SHGs doing to ensure sustainability?

Respondent 1: When we no longer receive support from NGO, they have already trained and taught us on how to seek support from other sources aside them. They are the district assembly, MPs, DCEs, etc. They trained us in how to seek support from these people. That we should write to them. So, when the NGOs no longer support us and we implement what they taught us, the group would be sustained.

Respondent 2: They also taught us that when a time comes and they are no longer giving us support, as a group, we can support one another. They gave us that training.

Respondent 3: Our group, when we no longer get support from those who are currently supporting us, they told us a time is coming when they will not be supporting us again. It is when you're a child that you suck your mother's breast milk but when you grow, they stop you. When they no longer support us, the chairs and canopies they have given us, as we rent them out to generate some income, we can buy more and add so it becomes a source of income for us when they no longer support.

Respondent 5: BasicNeeds taught us that as they are supporting us when they no longer support us what we think about it. When they gave us that training, for me I told them that as they are training us, like the saying goes, when someone is cleaning your front, you also clean the back. If not, one day you would suffer for it. I told them when they no longer support us with the knowledge we've gained through the trainings, we would implement that knowledge to keep the group.

Interviewer: Any other recommendation for effective SHGs operations.

Respondent 5: What I want to recommend is that I'm encouraging this group that we should unite. When we are united, it would help us a lot as a group.

Respondent 3: I'm pleading with everyone to be patient with one another. We shouldn't listen to gossip. This would help to continually move the group forward.

Respondent 2: we shouldn't listen to what people are saying. We shouldn't broadcast our challenges outside. We shouldn't also speak badly of the group to people. They might misinterpret us, and it can affect the progress of the group.

Respondent 4: We need patience. If we have patience, we can be together. May God give us patience.

Interviewer: I thank everyone for your time to participate in this discussion. On behalf of the researchers, I want to express our heartfelt gratitude to you. May God grant us good health and give us long life. Thank you all.

FOCUS GROUP DISCUSSION

Interviewer: My name is …… I came with my friends. My friends are called ……and ……. …….conduct this interviews but due to work schedules, he has asked us to represent him. So, we are representing……. We have a few questions to ask. Please, if you have the answer, you can mention your number before you talk. Now we are zooming into the questions. I will ask the questions in English and translate them to Dagbani, if you can answer in English, good. If you can't, reply in your language (mampruli or moar).

SHGs Operational Structures Within Communities

Interviewer: How do your SHGs operate within your community (ies)?

Respondent 4: Our SHG, how we operate, we meet once every month to advise one another on the progress of the group.

Respondent 1: What we also do in our SHG is that we visit those who are not able to come for the meeting.

Respondent 3: As we sit for the meeting, we learn a lot so that we can take care of ourselves at home. The bad things we could have fallen into we are able to avoid.

Respondent 2: As we meet, we also learn how to take care of ourselves, we are also able to encourage one another, and can take care of ourselves better

Respondent 6: Sometimes the sick person may say they want a particular type of food by force, you must plead with them until they understand you so everything will be peaceful that's what I also have to add.

Respondent 5: I may want a type of meal. If I can cook that meal, I cook for them to eat but if they don't want anything you don't cook for them.

Interviewer: Describe your typical day as someone with a mental health condition or disability to me.

Respondent 5: I can wake up and doesn't want to talk, don't talk to me or that's not it?

Interviewer: (**Interviewer** clarifies more on probes) How do you begin your work when day breaks from morning, how does your day go? **Interviewer** goes over probes (*for time work begins and ends, role (s) played, number of days on work (if any)*

Respondent 2: As a person with such a condition in your home, you cannot leave the home as early as everyone, you must be a little patient and delay to take care of your needs before leaving home. And when you're at work and it's time around 3pm – 4pm you must go home early to take care your needs.

Interviewer: Does anyone have something to add?

Respondent 4: when I'm about leaving home for work, I am a sick person so in the morning, I must check how they are faring first. I leave around 8am, because of the sickness I cannot keep long in the farm because being alone could be risky and dangerous, so I come back home from the farm by 3pm.

Interviewer: What are some of the pleasant experiences you have had as someone with a mental health condition or disability? (Translates into the local language)

Respondents: all ask for further clarification to the question

Interviewer: (throws more light on the question with the help of probes)

Respondent 2: What I will say as a pleasant experience is that I have had in this SHG is that when I come out and I don't feel like mingling with people but others want to so when I wants to mingle with people don't prevent me and don't me feel as I am no human. If there is a need to prevent me from mingling with a particular group of people, be patient explain to me and not make me feel it's because of the sickness, in that situation, I would be unhappy.

Interviewer: That's ok but you have deviated into a different question, this question has to do with a pleasant experience.

Respondent 3: The pleasant experience I would say is when I like a particular food and I eat it, I become happy, I have to get that food so that when I eats it I would be happy.

Respondent 1: When you give us the drugs the sickness is not able to disturb us much.

Respondent 4: Another good experience I see is that as you the sick takes the drugs, you become calm, as you are always hiding yourself and don't want to mingle with people and are able to chat with people for sometimes.

Interviewer: What are some of the unpleasant experiences you have had as someone with a mental health condition or disability?

Respondent 5: When people know you have this condition, they mock at you and do all manner of unpleasant things to you.

Respondent 6: There are some when they mock us, we begin to avoid the public and live in isolation

Respondent 4: what I also see which doesn't make me happy is that we like everybody but because of the sicknesses people especially those outside our families mock at our condition, saying we are 'falling-down', 'he is a mad person' and wouldn't want to come close to you that way it makes you angry and even the family.

Current Financial Operations of SHGs

Interviewer: Please tell me about operation of SHGs
What are your sources of funding?

Respondents 5: As we sit for this SHG meeting we do contributions to help the operations of the group. We contribute for a long period of time and when you do that, we use it to help one another that is when one of has a pressing need.

Interviewer: Which stakeholders have you been you been collaborating with to assist in your operations?

Respondents 2**:** It's BasicNeeds Ghana who come here frequently to collaborate with us for the past few years in areas of training and financial support.

Interviewer: Are you able to get support all the time when you need it?

Respondents 6: Yes, we get support because our 'susu' helps but it's not all the things we need that we are able to get.

Interviewer: When was the last time you had such support and who gave the support?

Respondents 1: It's been two years since we had support, and it was from social welfare

Interviewer: Anyone with anything to add? (No response) Alright let's move forward.

Interviewer: Collaborations with government institutions (district/municipal assembly, social welfare department and national health insurance scheme)

Please tell me about how you collaborate with governmental stakeholders in mental health care.

Respondent 4: We and government agencies are in talks. That is social welfare and the district assembly. We've written to them and asked for support from them. The letters have been acknowledged as received but the support hasn't come yet. From time to time, we've had meetings with them to remind them about our request when the means becomes available they should remember us so we would be able to get drugs.

Respondent 1: We also work with Ghana health service we have a psychiatric nurse who takes care of us. Also, health insurance renews our cards for us.

Interviewer: When was the last time you had such collaboration, who participated and where was it held?

Respondent 1: Last year, 2022, we met at district assembly with the social welfare department and health insurance manager in the collaboration

Interviewer: Any other thing?

Interviewer: When you met what were some of the key points discussed?

Respondent 1: When we met, we agreed that when the district assembly common fund is paid by government, we would be given 3% of the common fund and the health insurance department also said they would be renewing our health insurance cards for us when they expire.

Interviewer: Has the health insurance been able to renew your cards?

Respondents: (Chorus) Yes!

Interviewer: Any other addition? (**Respondent**s says nothing)

Community Involvement

Interviewer: How do you involve the community in the operations of SHGs activities?

Respondent 5: As we contribute the 'susu; money down and the way we assist one another it would motivate and encourage members of the community to want to join us.

Interviewer: Good, but I think you have not hit the question right on point as it demands, this would help you to understand better. For instance, when you are to engage someone, how are they consulted, is through written or phone call?

Respondent 4: This how we involve stakeholders and members to participate in our activities. There are some we go physically to invite; we sometimes invite the assembly member to come and have a talk with our members. We also invite doctors to come to us on how to take care of ourselves to the sickness doesn't go beyond our ability to bear. Sometimes we go to talk to them personally other times we call them on phone or write letters to involve them.

Interviewer: When do you usually call them and when you call them what do you engage them to do for you?

Respondent 2: There are times we call them when we have a meeting, and we need support from them. So, we request that they come so we could collaborate and plan the way forward for us.

Respondent 3: When they come, there are teachings they give us. They teach us that when you're the sick person and realise your disorder is to affect you, and there is no medicine readily available, you quickly rush to the hospital so you can access drugs for yourself.

Interviewer: Please tell me about any health campaign activity that you involved the community.

Respondent 4: we've had the nurses come talk to us and the community about metal health issues and we also brought forth our challenges, and they provided guidance on how to keep the sick people and on how to seek assistance from the nurses and from government.

Interviewer: But have you had a meeting where you gathered the whole community to educate them on mental health.

Respondent 1: We have ever gathered the community to educate them on the need to stop stigmatisation against persons with mental health conditions and disability.

Interviewer: What were the benefits and challenges you are uncounted.

Respondent 4: The challenge we encountered was that when we spoke with the nurses about the drugs, they said government has not supplied them with drugs and if you are someone with frequent seizures and goes to hospital and you are told there is no drugs, do not think that the drugs are there but they refuse to give you. So, you the patient would have to find out where drugs could be acquired so you go and buy so the nurses can treat your patient for you

Respondent 1: After we had the community health campaign, I have realised that there is a change. The rate of stigmatisation in the community has gone down drastically.

Interviewer: What were some of the unpleasant experiences you have trying to access health care as well as social protection avenues or opportunities?

Respondent 1: When we started and went to district assembly and ask for our 3% of the DCF they told us about that people with mental health condition and disability were not part of the beneficiaries of the fund. It's rather the physically challenged, not mental health patients and that did not make us happy.

Respondent 4: Just as number 1 said, we sent our names for help, district assembly told us they only know of widows and the physically challenged. But I think we the sick are more important but they refused but rather gave assistance to widows and the physically challenged that is what made us unhappy.

Respondent 2: When district assembly told us they only take care of widows and the blind and the physically challenged, we were sad. We are also people with challenges but why would they do selective assistance. It looked like they had no regard for our condition. We weren't happy.

Interviewer: Any other thing?

Respondent 3: I have ever been to hospital though it wasn't me, someone came with an epileptic who had seizures during other patients and people. When she fell everyone run away and left her I had to run and assist the care taker so we hold her and raise her. I fetched water for her to wash her up. This experience made me unhappy.

Facilitators and Barriers

Interviewer: What are the facilitators of SHGs activities?

Respondent 2: BasicNeeds Ghana have been visiting us to train us on how to take care of ourselves and our members. How we can leave together so that there would not be problems. When we have a challenge, how we can access support so that everyone can be happy.

Interviewer: Anyone with anything to add.

Respondent 4: When we started with BasicNeeds Ghana and they come to educate with us to train us how to take care of ourselves so that even when there is no longer support from anywhere, we should be able to be sought for support for ourselves

Respondent 1; When covid came, BasicNeeds came to gather us to train us how to take care of ourselves to avoid contacting the diseases.

Interviewer: But among yourselves what are you doing to facilitate your activities?

Respondent 2: We are also doing on our own as number 5 earlier mentioned is the 'susu.' So we are able to contribute for the 'susu' so in case one has a challenge or someone's medication has finished and they have no money to buy, we can go into our coffers to assist such fellow with

money to buy and when they get the money, he brings back and we put back into the coffers and when another has similar challenge we do same.

Interviewer: What are the barriers to SHGs activities?

Respondent 2 Our area of challenge is when you have someone to go but the sick person isn't very fine whatever you wanted to do, you wouldn't be able to do, if you wanted to go somewhere, you would not be able to go in that case it would disturb since you are not free to do your work as you wished to.

Respondent 1: Another challenge is the drugs. We don't get the drugs as we wish to.

Respondent 5: Another challenge we face is that if they could come to build a hospital or something for easy access to health care.

Respondent 4: Other challenge I see is that we have our own nurses and doctors. I this area I think our mental health workers are too few. We have only one such nurse. If they are more psychiatric nurses, it would have helped a lot. But one person all the way from Bunkpurugu to Nasuan. So sometimes you would need a doctor to take care of your sick and sometimes you have bought your drugs and injections, but they wouldn't be one.

Interviewer: What can be done to address the challenges people with mental health conditions face in this community?

Respondent 4: what I think when it's done would help a lot is that we would want to plead with health directorate and government to send us more psychiatric health workers to our area so anytime we need we can easily access them faster. And also, our drugs should be made available and accessible in our various hospitals.

Interviewer: Anyone with something to add?

Respondent 3: I think if we could unite to get our leaders so we can all take our challenges to DCE and MP, informing them that these are our challenges, and we need their assistance. That we lack nurses and lack drugs. they should try to assist us I think that would help to reach the government faster.

Interviewer: If anyone has something to add say it before we move to our last question for this session.

Interviewer: What role do you expect government agencies to play in meeting the health and social needs of people with mental health conditions?

Respondent 2: what I think government can do to help us patients is that the drugs are not available in the hospitals. So, since the drugs aren't available in the hospitals, there times the sickness would be disturbing you and your medications are finished that's when you begin running around to get the drugs. So, if the government did her best to ensure that the drugs are available in our hospitals so when you have a problem and you run to the hospital you would get drugs.

Respondent 5: That is why I said before that if we had our own hospital so the medicine can be there so when you have seizures or your medications are finished, you can easily go for your medication at the hospital and go back home.

Interviewer: (clarifies) so you wish the government would build mental health condition and disability hospitals?

Respondent 5: Yes, yes!

Recommendations and Sustainability

Interviewer: What is your SHGs doing to ensure sustainability?

Respondent 4: what we are working on so that in case BasicNeeds Ghana ceases to give us support either today or tomorrow. We have started a 'susu' contribution. We would also want that as of now, there could be support given us in the form of livestock or birds so that we begin rearing so the day BasicNeeds would no longer give us support, we would be able to survive or such help could come from government so the day BasicNeeds can no longer support us we can depend on it and our susu to survive.

Respondent 1: It is also good we meet frequently.

Interviewer: Anything to add.

Respondent 2: As 1 mentioned about our meetings. If we are meeting frequently, the day Basic Needs would no longer support us and we continue to meet to encourage one another it would help to sustain the group.

Interviewer: Any other recommendation for an effective SHGs operations.

Respondent 4: To me, I think as we meet frequently, we should agree so during the farming season, we can go for by-day farming work so we can get more funds to add to our coffers.

Respondent 6; Also, during groundnut harvesting, we can go to work to make money to assist the coffers.

Respondent 3: There are a lot of work I think if we have the energy and ability, we could also go for groundnut uprooting or maize harvesting. That is what I also think.

Respondent 4: I think that if we go introduce ourselves to the chief and he is aware about this category of people has a group in his community. Anytime we need help or support, he can go on our behalf to seek for support on our behalf. If we inform the chief and receive his blessings, it will help us.

Interviewer: We want to thank you all so much for your time, I would stand on behalf the researcher I wish to thank you so that lets continue to be united and may the Lord bless help us all.

FOCUS GROUP DISCUSSION

Interviewers: Good morning to you all. My name is …….. My name is ……. I'm ……. These are my colleagues from ……We have come and take this information from you and he has also sent us to stand on his behalf to take this information. We will not use your names; we'll give you numbers. When you are to respond to a question, mention your number and respond to the question. When I ask a question, any of you can raise your hand, mention your number, and give your response. When someone speaks and you have something to add, mention your number and add what you have to say. You would be number 1, my sister would be number 2, my uncle would be number 3, you would be number 4, then number 5 and number 6. I want us to move straight to our questions.

Interviewer: First, how does your SHGs operate within your community (ies)? (probes added)

Respondent 1: We started by meeting every Sunday without a miss, that was how we began. But we continued for a while, there were challenges, so we began meeting first Sunday of every month until we again realized members were forgetting our meeting days Even I myself was a victim and we met again and came to a conscientious that we would meet first and last week of every month so every month we meet twice.

Interviewer: Any addition

Respondent 5: As 1 said it's true, we used to forget the meeting days now we have rearranged it. And we come we greet one another to know how everyone is faring, check the number of people who came for the meeting, and then we sit to hold our meeting.

Interviewer: How are resources sourced for the group

Respondent 2: For resources, we make contributions of one 1 cedi. It can happen that your medications are finished, and you go to the hospital and they write for you to go and buy Even if your money isn't up you can borrow some money to go acquire some medications while you continue the 1 cedi contributions.

Interviewer: Any addition

Interviewer: Describe your typical day as someone with a mental health condition or disability to me.

Respondent 1: Based on my body condition, when day breaks…

Interviewer: (interjects) I mean your work routine in a day…

Respondent 1: Yes, I will get there, every morning based on my body condition I think about the work I would do concerning my health. I think that I can make money to keep myself so in case I have any challenge, I can use it to assist myself, that is my thinking.

Interviewer(s): What we want to know is when the day breaks, what you do from morning to evening, the time you go to work, the time you come back from work, etc. as someone with a mental health condition or disability.

Respondent 5: Because we are not really well, we are not able to go to work as everyone. You can go to work and seizures can distract you and you wouldn't be able to work. Unless you are patient with yourself, work small for the day and then the day you are very strong you go to work.

Interviewer: 2 was to add something

Respondent 2 Sometimes when the day breaks, it is not every day you would be healthy and strong enough to go to work, the time you go to work, there are times there are people at our work sites who stigmatize us and discriminate against us so something happens, they shout and call you mad person. This makes me feel relaxed to go to work but when you go to work and you are well received by your family, your mind would be at work and every morning you would be enthused to go to work. So, there is no peace of mind as you wouldn't have the desire to go to work.

Interviewer: Any other, Number 3 you are yet to speak. Ok, let's continue.

Interviewer: What are some of the pleasant experiences you have had as someone with a mental health condition or disability?

Respondent 1: It usually happens sometimes I can meet someone I've not seen for a while and He asks how am doing, He encourages me by telling me I will be fine. When that happens, I feel happy because someone cares for me, and I feel someone empathizes with me. So, it gives me joy and happiness knowing that I am considered part of society.

Respondent 4: Sometimes if I go to meetings am always comfortable because I can look left and right and know that this is my colleagues but if am in the house, I will feel lonely.

Interviewer: That's good. Number 6 you've not said anything yet or we should move forward. Who else has had a pleasant experience? Number 1 spoke, and Number 6 too. Now let me ask this question.

Interviewer: What are some of the unpleasant experiences you have had as someone with a mental health condition or disability?

Respondent 1: It often occurs that you will be among your peers to do something. When something happens, they make derogatory comments about you. When that happens, you will not be happy because you are not expecting people to stigmatize you in that manner. So when that happens and you are at the place at that time is always a challenge to you because how bad am I that people don't want to relate with me?

Respondent 6: What they said is true because the conditions wouldn't allow us to live our normal lives, sometimes you travel to different communities because of our condition we get worried that you may get attacked, and if it eventually happens what you were there for you wouldn't be able to do it, after that all mind will be unstable and because of that you may have to return home, you may go with the condition to any community and before you arrive you get the attack, if its

mentally challenged you will struggle and those you were going to work with because of the experience they begin to stigmatize you, so is a lot of thinking for us,

Respondent 2: It may happen because of our condition, assuming they send you for an apprenticeship and it happens that the day you arrive, and you had the attack, everyone's attention comes on you even if you are given a task none comes close to you again and because of that you are not able to freely stay and learn the work.

Interviewer: is asking for more experiences

Respondent 5; It has never happened even if you can go to the hospital to collect the medications and if the attack comes you will see the nurses take you into an isolated room until you regain your full consciousness.

Interviewer: Number 3 we are still expecting your contribution

Interviewer: Current financial operations of the group and our first question is where you get monies to sustain this group and where do you get them from,

Respondent 3; Truly as number 2 earlier contributed, when we attend meetings, we make contributions one, one cedi down If it happens that one of us has no money and his medications are finished, he should come and borrow, I think when we started this has been our source of money, but we do get help from NGOs as you have come.

The **Interviewer:** is asking for more clarity on which NGOs specifically came to and the kind of support.

Respondent 3; like Gupkatimali has never come and given us help and basic needs Ghana has never come and given us support, this is where we get our support from.

Interviewer: I want to ask how the help comes, like Can you describe in a year how many times you receive them?

Respondent 4; The help we have been getting is not frequent it can take one year to four years at the time it comes occasionally.

Interviewer: so, can you remember the last support you had and from which organizations

Respondent 2: 2020, In the year 2020 we received support from basic needs Ghana, they came and helped us with tailors and also sponsored our apprenticeship.

Interviewer; So, is that the last support? that is very good, Does anybody have anything to add so I can continue with the questions?

Respondent one: after receiving that support in 2020,2021gupkatimali also brought support if only is permitted to mention it then they brought us foes dresses and some bags to we who are not well, they were small backs that you can give to your children to go to school among them were others like purse and some shirts even thou they weren't bad dresses, I think that was the last time basic needs Ghana gave us support that was in 2021.

Respondent 4: Last year that was 2022, we also celebrated our mental health day, and then we got some support from Songtaba to help us buy water for our clients.

Interviewer: that's very good let's continue, we going to discuss your Collaborations with government institutions (district/municipal assembly, social welfare department and national health insurance scheme)

Respondent 1: we had a meeting with health insurance on that day we called the social welfare, the health insurance director, and the assembly the meeting took place at the health insurance, on that day the psychiatry nurse in charge of us was also present social welfare director was present, the insurance director was present. The one who came, and we organized the meeting was Mr. Adam from Basic Needs Ghana. He brought to our knowledge that if we are not well that is we the clients, he told us that health insurance renews our cards free of charge or any new without the health insurance card they offer such members too help to acquire the card and they also told us we have some support from the district assembly, I think we have given assembly letters several times, they informed us that truly our support is with them so we should choose among our self's some for support, also we met and we mentioned our challenges which is our medications, we wrote letters to the district assembly and social welfare. just that we didn't get any help aside they are reassuring us, but this year helped one of us and encouraged us to be patient if we get to the coming year, they will see to it that we will receive their support that we are not the only group so we should bear with them in patience.

Interviewer: is asking about the last meeting month with them

Respondent 1: I think the last meeting we had informed us; they have finished the support they have unless next year was two months ago this year. I went and told them within 2023 that I wanted to know because we had a challenge with them, they said that it is only the blind and the physically challenged that they want to give support. When I went there, I wanted to find out the number of us they said they would support because they would want to know our total number before, they would be able to know the amount of support they could give us. So, we met with them and they said we would to know the number of us who haven't received support from them so they can plan how best to support them so this year we met with them.

Interviewer: Moving forward, how do you involve the community in the operations of SHGs activities?

Respondent 3: Yes, we involve the community in our activities. Anytime there is any gathering, and they need people to come we also get involved, for example, in clean-up exercises, we also get involved so we can ensure the cleanliness of our environment.

Respondent 4: when we were celebrating our mental health day, we invited the assemblymen who are the mouth to the community so they can also talk about the stigmatization.

Interviewer: Please tell me about any health campaign activity that you involved the community.

Respondent 1: Yes, on World Mental Health Day, we gathered a lot of people, as 4 said, we had the assembly members and community leaders.

Interviewer: What were the benefits and challenges you faced?

Respondent 1: We made the public know it seems they are discriminating against us and we the sick even in our families when something is happening, they talk to us in a way that doesn't make us happy because when you do something and they aren't supposed to say some things, they say them to you. We were able to educate them that we're a part of them, and whatever that is happening to us they are supposed to give us peace of mind thus we need their support even if they cannot give us financial support, they should be able to inform us about where we can get medical support. All these we educated the public.

Respondent 6: To add to what 1 said, in our zone when you go to register people with mental health challenges, because they are stigmatized, they are usually not willing to make their condition known.

Interviewer: We would want to know the challenges you encountered during the health campaign

Respondent 4: We also educated the people on how to come out because if you are hiding in the room and there is a problem we cannot help like sometimes someone would look at you because you are sick, they can impregnate you and leave you, that one we talked about it and the community leaders gave us a number that in case of any such issue, we should call and they would arrest those people because they are infringing on our rights.

Interviewer: Any other thing before we proceed

Respondent 3: As 6 said, sometimes it can happen because they stigmatize, some of them hide, and some families even lock them up in chains and rooms, during the campaign we made them to understand that we are also human beings, and when we come out to meet our colleagues, it gives us peace of mind but they are locked up, it makes them to think a lot but when they come out as we are out it would encourage a lot of them to own but most of them are not owning up.

Respondent 5: I think the reason they lock some of them up is because some of them can come out and run into cars, or gutters would be dangerous for their health that's why I think some people lock them up.

Interviewer: Have you had any unpleasant experiences while trying to access health care and social protection avenues or opportunities?

Respondent 1: Yes that happens, you can go to the hospital and some of the nurses can stigmatise you because of epilepsy or mental disability. They won't give you the needed attention. But as for the psychiatric nurses, they are better. None of them has ever done us anything that makes me unhappy. For them, anytime you go they have time for you but for the other nurses, they can treat you in a way that makes you feel you are not a human being.

Interviewer: Anyone with such experience also.

Respondent 4: Even the patients who are sick and they come to the hospital, they also stigmatise saying that are you looking for your mental health nurse, go and look for them this not the place to look for them.

Interviewer: Any of with such an experience also?

Respondent 6: That is there, and it is everywhere. Because you have the sickness, you know, not everyone has common sense. When you go to hospital some of the people would treat you any how and you look at your life and feel so sad, but it is God who has allowed that to happen to you. So, it exist.

Interviewer: have you also had any pleasant experiences while accessing health care?

Respondent 5: There are pleasant experiences. I have been to the hospital here to take the medicine, and the nurse was very caring. The nurse asked how I was faring and treated me kindly and I was very happy.

Interviewer: Any other person with such an experience?

Respondent 1: I have never gone to take my medication. I came across a colleague who was brought from Gbintiri. When he came out to go home with the person who came with him, I was so happy the way the nurse received them and the caretaker was also so happy because the moment they came, the nurse said she has realized that as the patient is taking the drugs he has changed completely and, but he has added weight and what is the reason. He was mentally disabled, and the patient responded he has been eating a lot of eggs. When he finds people eggs, he picks them and eat, and the nurse replied that we are supposed to eat one egg per day but when you eat too much it can have negative effects on you. And the patient replied, "Is that so?" then the nurse said yes. Then the nurse told the patient to take meat and eggs in moderation. When we came out the mentally disabled person said he likes the nurse but not taking plenty eggs and meat is tough. I was so happy the way the nurse received them.

Interviewer: What are facilitators of SHGs activities?

Respondent 4: Anytime we have our self-help group meeting, it is good for the psychiatric nurse or mental health nurse to be there. Sometimes too it is good for the social welfare to be there so that we will also feel like we are part of the world.

Respondent 5: When it comes to the facilitators, I think if we get livestock to rear it would also help to occupy us and keep us happy.

Respondent 6: What can also facilitate our activities is when we get skilled work, it would help us to have peace of mind, and you can go to the work every day so you can also be a part of life.

Interviewer: Anyone with any other thing to add?

Respondent 4: If we also get finance from other NGOs to start business because most of us are not working. So, we also start small business or rearing of animals

Interviewer: Number 3 anything to add?

Respondent 3: Ok to my opinion, those who come to visit and talk to us about our welfare, I think if they could get assistance for us. There some of us when we take the medication and we are fine, when you are fine but has no work to do, if we could get some financial support, we could use to buy toffee and biscuits to sell so that we will be able to get money from the business to feed because hunger is one thing that makes our sickness more serious.

Interviewer: Barriers to the activities of the group.

RESPONDENT 1: If it happens that we have a lot of petty quarrels or lack of concern by members. You did not see someone in a meeting, and you don't visit to know why they weren't at meeting this week. When you follow up on the person, and their relatives see that, these people come to check up on their colleague who wasn't at meeting to know what is wrong with them. Because of that we go for house-to-house visitations. We don't joke about it. We do it a lot. Even when our days of meeting is not up, still we visit our members so that they would know we have so much concern for one another

Respondent 3: What I would add is that it can happen that you sit for a meeting and wants to come up with ideas that would help the group to move forward. Someone can come up with an idea and when that happens, and you want to say that what one person is saying cannot work but the other person's contribution doesn't make sense. It can make the person feel you are discriminating against them, or you are demeaning them. You must take both suggestions and say that what both have said is good and then look at the two ideas and see how best you could modify it to make it workable.

Interviewer: What role do you expect government agencies play in meeting the health and social needs of people with mental health condition?

Respondent 1: You have brought a very important issue up. That has been our major challenge. We need drugs. The drugs is our main issue we want government to address it so we could be getting the drugs and not this go-and-buy, go-and-buy. That each time we need the drugs government would help to ensure that it is available in the hospitals, so we go pick. All these would help to promote our health.

Secondly, we spoke of support as number 3 mentioned earlier, when day breaks and you are fine but has nothing to do it is also a sickness. So, we would need the drugs and support that can transform our livelihood.

Respondent 6: Another support we would need is that in some of our zones when you go to our hospitals you wouldn't find them as we need. They don't care for us there. Even you could help to advocate for us so that we the patients would know that as we are there, they fight for us. In our zones that doesn't exist. So if the government could take a look at it, it would have been so helpful to us.

Respondent 3: What I also see that they should do for us is that our nurse is only one because he is only one, government could try its best to increase the number to two or three. So when someone isn't well and they cannot carry the patient to the hospital, because he is the only one, when he goes, no one is there again but when he goes and there is one more nurse left at the

hospital so the one who is also able to come to the hospital can receive treatment. So if they could it.

Respondent 4: More to the point if a person relapses. It is good the person should get injection but sometimes it is only the drugs that is there and that one is not even at the clinic. You have to go and buy. Sometimes you go to the drugs store, some would not be there. You have to be there like that.

Respondent 6: Like madam said, it happened to me. This is the number 6 speaking. When it happened to me one day, it was the police who did their best to assist me. They picked me up and laid me at the police station for sometimes before my people came for me but it isn't the job of the police. If they were there, wouldn't they have helped?

Interviewer: What is your SHGs doing to ensure sustainability?

Respondent 4: They educated us that we have a common fund at the district assembly. We should always apply every year or a year we should apply twice. We have been applying but we don't get anything. This year, only one person received support from them. That's the place they told us that's our mother place. We should always go there for any help that we want

Respondent 1: What we can do in addition is that those of us who have been given sewing machines, driers, and animals, when a time comes and we don't get support from anywhere, at that time, it would mean that we ourselves should come out to work on our own even if it means we contribute. For instance if six of us are tailors, and four have livestock and five are hairdressers, when we meet, because of the unity, we can take a decision to be contributing money down so that anytime we have any problem we can use that money to sought it out. So if we are able to get support in these areas I have mentioned, because of our unity, we would be to sustain the group so that it can move forward.

Interviewer: Any other Recommendations for effective SHGs operations.

Respondent 1: The way you have come is a very good thing for effective SHGs operations. They would know that not only we those who are not well are concerned for one another but there are people elsewhere who have special concern for SHGs for its existence. When it even happens that we say visitors are coming to meet us, all these are ways that boost our morale and those who are not members would usually want to be part of the SHG.

Respondent 4: The education we receive from the NGOs is also building our efforts on the groups because it means that without those people the groups wouldn't have existed.

Respondent 5: What I would add is that we should encourage one another to take the medications. When you go to the hospital to take the drugs, you have to take it.

Interviewer: We are most grateful for your time, may the Lord help us. When opportunity presents itself and we meet again, we will meet you in good health. Thank you so much.

(HAND CLAP)

FOCUS GROUP DISCUSSION

Interviewers: Good morning to you all. How is home? And your family? We thank God. My name is…….. We have come and taken this information from you and he has also sent us to stand on his behalf to take this information. We have questions for you but before I start asking the questions, we don't want anyone to mention or call his/her name. So, I'm going to give you numbers when you get up to answer the question you first of all mention your number and now answer. so, uncle, you are #1, grandfather you are #2, Mommy will be #3, the next number 4, Mommy you will be #5, and finally grandfather, you'll be #6. I hope we have understood what I said. If you can't pronounce your number in English just pronounce it in Dagbani, I hope you all understand. I also want to plea that when you are about to talk, please speak louder so that our recorders can pick it up.

Respondent: yes

Interviewer: OK so my first question is

Interviewer: How do your SHGs operate within your community (ies)? · **Probe** for times/days of operation, membership, convening of meetings, frequency, participation during meetings, how resources are sourced, etc.

Respondent 6: This is how we have our meetings; in a month we meet once and that is always at the end of the month the last Saturday or Sunday of the month. That is how we meet.

Interviewer: Any other addition

Respondent 2: This is how we usually meet; we meet twice every week and that is on Friday and Sunday.

Interviewer: OK any other addition

Interviewer: Describe your typical day as someone with a mental health condition or disability to me. ***Probe*** *for time work begins and ends, role (s) played, number of days on work (if any)*

Respondent 5: when day breaks it's not every day that you feel very well there are some days to be OK some days to you know be OK and so even if you have somewhere to go you can't go unless you are OK that's when you can move out and know whether the work you are doing you will be able to work today.

Interviewer: So now that you are OK what time do you always go to work and what work do you do?

Respondent 5: In the morning when I wake up at 6:00 am, I move out to set fire for my pancake business, if I'm feeling fine, I will sell for a while but if I'm not feeling fine I'll close early.

Interviewer: Any other opinion?

Respondent 2: For me, my help is BasicNeeds, Gubkatimali, and district assembly they help me to farm. I farm groundnuts and maize. My work is just to farm groundnuts and maize.

Interviewer: OK so when the day breaks what time do you go? And what time do you come back?

Respondent 2: When the day breaks and I go to the farm, I close around 12 noon and now come home to rest so that I can go back the next day.

Respondent 3: For me, I don't have any trade. The only work I do is, go and help Nba Lawyer grind meals. That's where I am working. We are helping those who come to grind their meals so when they come, and we help them at the end of the day do get us something small so that we can share among ourselves and we are 8 in number Because we are eight sometimes I don't go early sometimes I go around 12 noon or 1:00 PM and I will be there till evening before I go home.

Interviewer: That’s good I'm happy with the way you are answering the questions. Now let's proceed.

Interviewer: What are some of the unpleasant experiences you have had as someone with a mental health condition or disability? How has being a member of SHGs contributed to this experience in this community?

Respondent 6: The unpleasant experience I had was on the day of voting, everyone was in the queue, and I came and joined. I was in the queue when my sickness was triggered, everyone ran and left me alone over there and when I woke up, I realized I was alone over there I really felt bad so, I left the place to the house.

Respondent 2: The most unpleasant experience I ever had was when BasicNeeds gathered us for a meeting to give us drugs here in this town. I came to the meeting and fell to the ground because I was dizzy and they took me to the Buntanga hospital, that's the most unpleasant experience I ever had.

Respondent 1: For me, it was in school, It was Friday and we were in class and the sickness got triggered All my classmates ran out and left me. I really felt bad that day and that was what took me out of school up to now I have not gone back. I now went and learned a skill. I wasn't happy about it at all.

Respondent 4: I went to play football, and I was playing with my friends when the sickness got triggered, I was down, the time I regained consciousness I didn't see any of my friends again.

Interviewer: That’s good I'm happy with the way you are answering the questions. Now let's proceed.

Interviewer: What are some of the pleasant experiences you have had as someone with a mental health condition or disability? How has being a member of SHGs contributed to this experience in this community?

Respondent 6: What made me very happy was, in 2015 that time the DC for this district helped us with some money which was 5000 cedi, and it was used to buy medicine for our office. Also, Gubkatimali and BasicNeeds Ghana also supported us with some money, and we used it to develop ourselves. Up to now, we are still benefiting from it.

Respondent 2: What made me happy was when our DC called us and gave us 4 sheep each to rear. It made me happy. BasicNeeds and Gubkatimali also called me and gave me something small and that was 6 (six) years ago they supported us in farming. That was what made me happy.

Respondent: What made me happy was that Gubkatimali and BasicNeeds came and gave us clothes.

Interviewer: Can you me the year or the period

Respondent: That would be like 4 years ago. They gave us money before they came back to give us clothes. It was during that period we were excited. And since then, we haven't heard anything again.

Respondent 1: BasicNeeds and Gubkatimali came and gave us clothes and it made me happy, they also gave us 400 cedis to buy life stock, after buying and rearing them they have now multiplied. This is what made me happy.

Interviewer: Great, May God let it multiply more. Amen. Now let's continue.

Interviewer: Please tell me about the operation of SHGs

·**Probe**: sources of funds, funding stakeholders, frequency of receiving support, last time support of received, and by whom.

Respondent 5: Is not all the time we get funds or support, but it comes periodically Nonetheless BasicNeeds came and gave us 300 cedis each and it benefitted us well.

Interviewer: Can you me the year or the period?

Respondent 5: For about 5 years now

Respondent 6: Where we usually get support from is, sometimes within this group. We usually contribute money any time we meet and it has helped me, I have people here who can testify that when BasicNeeds came and gave us COVID-19 drugs and preventive stuff (Nose mask, hand sanitizer) and it got finished we used the money we had in our account to continue buying the sanitizers and the hand wash, so anytime we have met before we sit for the meeting we wash our hands first.

Respondent 2: We also make small contributions in our group. We received support from BasicNeeds too. Sungtaba also came and gave us some support.

Interviewer: What support did you get from Sungtaba?

Respondent 2: They come every 3 months and when they come, they give us an amount of money for 3 months before they come again.

Interviewer: Please let's all bring our focus here so that we can answer the questions well. Now let's continue.

Interviewer: Collaborations with government institutions (district/municipal assembly, social welfare department, and national health insurance scheme)

Respondent 6: District assembly is those we usually collaborate with, I remember 2-3 months ago our doctor called me to help social welfare fill out some documents with our details, They said they were going to send them to their office, but we haven't heard anything from them again even duo we keep in touch.

Interviewer: Which period was that?

Respondent 6: 2 months ago or may not even reach 2 months.

Interviewer: OK, any other addition? Tell me about the frequency of meeting with institutions (district assembly, social welfare department, health insurance)

Respondent 5: Some time ago health insurance came and renewed our cards for us. That's what I'm even using currently.

Interviewer: OK, any other addition

Respondent 2: We used to work with health insurance scheme and I'm still using their card too.

Interviewer: OK, any other addition?

Interviewer: If there is no other addition let's continue. I hope I have your attention.

Respondence: Yes, we are focused. You can continue.

Interviewer: OK, now to my next question. Please tell me about how you collaborate with governmental stakeholders in mental health care. **Probe** on the last time such a collaboration was held, where, and who participated.

Respondent 6: How we usually meet our doctor. Every day our doctor is always at his office, but the medication always varies. Sometimes he can give you 3 weeks of medication, sometimes 2 weeks or even a month's medication. So, every day the medication varies depending on your condition. So, He is always at his office anytime your medicine finishes and you go you will meet him, so for me, the doctor is always available and is good for us.

Interviewer: OK, not only the doctors. How about the district assembly? How many times have they come for meetings; even social welfare how many times have they attended your meeting?

Respondent 2: Our doctor always gives us the medicine. Gubkatimali also supports me.

Interviewer: OK, any addition? When did BasicNeeds come here? How many times has BasicNeeds come here? How many times have they supported you?

Respondent 3: Truly I used to go to the hospital for the drugs but now I don't pick drugs there. Sometimes I will let my brother send me to Tamale so, that I can get an injection there. But now Truly I'm fine because the way it used to be, now is not like that again.

Interviewer: OK, why don't you go for drugs in the hospital again?

Respondent 3: I used to go for the drugs, but they told me to go to Gurugu and get an injection so now I usually go to Gurugu for the injection.

Interviewer: OK, let's talk about district assembly. Have you gone to the district assembly before? What was the discussion?

Respondent 2: I want to still talk about BasicNeeds because they are those who always come here. We are always in touch with BasicNeeds all the time.

Interviewer: OK, when was the last time they came, and what was the discussion about

Respondent 2: Ok that was last year. They gathered us and taught us how to handle and live with a mentally challenged person. They trained both the caregivers and the patients**.**

Respondent 5: Yes, they came, any time we hear of a meeting is BasicNeeds, no other organization has called us for a meeting, they and Gubkatimali always gather us, and any time they gather us they train us and encourage us to always come for meetings.

Interviewer: OK, to my next question. Tell me about the skill training they gave you to help you financially.

Respondent 6: The skill training we had was, first they gave us money and we bought life stock out of the money and used some to farm, they later came and inspected my harvest in my house, and they took some pictures of the farm produce. I still have evidence of their visit.

Respondent 5: What I witnessed was they told us to try and keep our privilege fund active. What I have experienced in this condition is, I went and sat somewhere and was just talking loosely and someone said look at how I went and mixed up with some people. It didn’t make me happy.

Interviewer: OK tell us about the skill they have ever trained you on

Respondent 2: Gupcatimali they came and gave me Gh₵600 to be used for farming and is about 4 years now even Mr. Adam from (Basic Needs Ghana) came and took a picture of it.

Interviewer: Calling for more responses

Respondent 1: They gave me 400 cedis, and I used it to buy two goats for rearing as I speak, they have increased in numbers, and it also offers support to the group.

Respondent 4: They gave us Gh400, and we bought 4 goats, Unfortunately, they stole some leaving only one.

Respondent 3: They also gave us GH400 each, then my husband took it and bought two sheep. One died and the other is missing.

Interviewer: The support is many indeed. Now to my next question. What skill training do you want in this group?

Respondent 5: We want money to do a trade because we sell a lot of stuff, Also A Sickler cannot save money because the sickness will consume all the money so because we are in to trade the money is not stable, so we need a little push.

Respondent 2: What we need from you people is I am a farmer and also rear animals so I will need money support so that I can farm well and also take care of the animals.

Respondent 1: Yes, I am skilled already. I repair ceiling fans but due to the lack of money, if you want to venture into a different business, you can't.

Respondent 6: For me the skill I think that would perfectly fit as is as you know for a Sickler all the time you have to be washing, so I'm begging if they can help us and teach us how to make soap. For the soap making I think everyone in this group would benefit from it. if you know how to make soap, you can make and sell and use it yourself. So I think soap making will be best for us.

Interviewer: Calling for more responses

Respondent 5: Soap is our main issue because when sickness triggers, we usually fall, and our clothes get dirty. So why don't we know how to make soap, we can use it ourselves and also sell it.

Interviewer: Let's move forward and leave the soap.

Interviewer: How do you involve the community in the operations of SHGs activities? **Probe** for who in the community is engaged, how are they consulted/involved, when are they engaged, what roles the community members play, etc.

Respondent 6: How we are involved in meetings with our community, or the town is sometimes when there is a meeting and we are part of the meeting, they do announcements in the mosques, churches, and radio stations (simili radio). They usually do that and tell us about the agenda of the meeting and what we are also supposed to do sometimes the agenda can be on sanitation and when we join we do go around and keep our community clean.

Interviewer: Any more contributions?

Respondent 2: This is how we get into our society, The radio stations help, and also, we those in the villages through the gongon beater. The gongon beater will give us that information about the meeting. And this is how we get involved.

Interviewer: Thanks, and let's continue. Please pay attention to the discussion.

Interviewer: Please tell me about any health campaign activity that you involved the community. **Probe** for names of health campaign, when it was implemented, for how long it was implemented, what were the challenges, and what were the benefits.

Respondent 6: Yes, we have some in this community. Some time ago we had one durbar at the secondary school in this community, the country director was in the meeting, a representative of our chief was there, and all SHGs in this district were present.

Interviewer: When did you have this campaign?

Respondent 6: It has been a while. Many of us were allowed to advocate and let the people know that the condition was not transferable. So, if you are with someone and the scissures start, you don’t have to run and leave him, you can help him to lie down for a few minutes it will be over. Unlike those who thought when the sick flush Nate and you are close by you will acquire the disease, they were sensitized it that is not so.

Interviewer: What were some challenges organizing it?

Respondent 6: There were many challenges since it was more about the sick It was difficult without funds even to the extent of having this dignitary, we had to source for help from imams and pastors, and the DCE Assembly members helped us with some canopies and chairs for us to have a successful campaign.

Interviewer: What are some of the things you think if we do the group will progress?

Respondent 2: What can make this group progress is money. Our main problem is money.

Interviewer: Money to do what specifically?

Respondent 2: To help the group.

Respondent 6: If they can give us skill training it will help in our well-being because they can’t be giving us money all the time. But if we are working, we can get money through our work.

Respondent 4: We plead that you help us with the skill training. Training like tailoring, carpentry, Maison, or any other training that will benefit us.

Respondent 3: On the skill training this is my view, if is soap making, you will be able to do it very well but if it is any other skill some of us cannot. To me, I think is only the soap-making some of us can do.

Interviewer: What are the facilitators of SHGs activities?

Respondent 1: I think if they help us with the soap-making, it will help this SHG.

Interviewer: Is it only soap? Bring out more views!

Respondent 5: If you give us money, we will know what to use it for so that it can generate income for the SHG. You can follow up for evidence of the income that it would generate since you want proof. So we just need some morning so that we will know where to invest it in.

Interviewer: What are the barriers to SHGs activities?

Respondent 6: What will make this group dissolve is when we don’t attend meetings, If we sit to discuss it will help the group to grow but if we meet always we will be able to discuss the way forward and also keep in touch with each other.

Respondent 1: That’s true if we attend meetings this group will grow but if we don't this group will die off.

Respondent 2: Meeting attendance is very important because we are from different places and is the meeting that will help us know each other.

Respondent 3: Truly attendance at meetings will help us know each other better but if we don't meet always, we will not know the way forward.

Interviewer: What are your challenges?

Respondent 5: When we meet, also try to visit us regularly it will help encourage us and also encourage you as you know we are doing our best. Anytime you call us we will come.

Respondent 6: What I want to add is everything in this group is centered on taking drugs. These days getting the drug it's difficult and if you don't get the drug, we will not be able to come to meetings because we won't be in good shape.

Interviewer: OK let's continue. Please hold her so she doesn’t fall (one had the attack). Please pay attention.

Interviewer: What role do you expect government agencies to play in meeting the health and social needs of people with mental health conditions?

Respondent 6: What we want the government to do is, we don’t have a mental Health facility around, so we appeal to the government to help us.

Respondent 2: The hospital is our main problem.

Interviewer: What do want the government to help you with?

Respondent 4: I will say that drugs there are days that you go to the hospital and the drugs are not there, you have to go and buy them and if you don't have money to buy that would be a problem. So, the government should help make that drug available in the hospital.

Interviewer: We are edging closer to the end of this interview now to my last question. What are your SHGs doing to ensure sustainability?

Respondent 6: The skill training we talked about is what will help us to sustain the group. Is the skill that will help us get small money to support the group.

Interviewer: Any additions? What do you think you can do to sustain the group if you no longer get support from the government?

Respondent 4: We are appealing to the government to help us with the skill so that when he no longer supports us we can support ourselves to get that drugs.

Respondent 1: Actually, some have the skill but others don’t so I think those who don’t have if they are also trained it will help this SHG.

Respondent 2: The skill training is a good suggestion so if many are skilled our groups will not dissolve.

Interviewer: What are other recommendations for effective SHGs operations?

Respondent 6: Like we said others are hidden so if the government will give us a mental health facility it will help bring many out to take their drugs and it will show that we are passionate about each other.

Interviewer: Any other additions

Respondent 5; Like my brother said to grow you would have to help us in business. As we attend our meetings, we may meet someone and the person will ask when is our next meeting again, so Because we meet it helps us create that scene of relationship with each other, so if there is no support and we are not able to meet then the group will die off.

Interviewer: I want to thank everyone for the time and for sharing your ideas with us. First of all, I want to thank you, and may the lord bless you, I want to stand on behalf of the Basic Needs Ghana and Dr. Phillip to thank you. May God help you and grant you a speedy recovery.

Respondent: I also stand on behalf of this group to say thank you to basic needs for always keeping us and supporting us God bless and keep you. When we were home with the sickness, we didn't know what to do but when you came and showed us how to handle it, we saw changes God richly bless us all. Amen.
