# Supplementary material for: Does supporting self-help groups of people with mental conditions for longer duration lead to more effective groups? A qualitative evaluation in Ghana
Source: PLOS Ment Health. 2025 Jan 30;2(1):e0000249. doi: 10.1371/journal.pmen.0000249 (PMC12798605; doi:10.1371/journal.pmen.0000249)
Supplement: Attachment — Submitted filename: Response to SHG Comments.docx [file pmen.0000249.s002.docx]

**RESPIONSE TO REVIEW COMMENTS**

**RESPONSE TO REVIEWER 1**

1. The article is a well written scientific article. As the authors have used the RE-AIM framework in analysing the results, I believe that the discussion of the results should be presented in the context of the RE-AIM framework. Further, there seems to be large amount of data that has been collected, however the amount of data does not get reflected in the paper and data underlying the findings has not been attached as a supplement. It would be good to have a conceptual framework at the end of the results to understand how the RE-AIM framework was used to understand the results. I suggest major revisions for this paper
1. **Response:** Thank you for the comments. A table has been provided at the beginning of the results to summarise the main and sub-themes based on the constructs of the RE-AIM model that was adopted for this evaluation. A description of the RE-AIM has been provided under the study design.

**RESPONSE TO REVIEWER 2**

Reviewer #2: Thank you for this manuscript on an important topic that presents a qualitative evaluation on whether supporting Self-Help Groups of people with mental conditions for longer duration leads to more effective groups.

2. Page 2-3 lines 91-97
   It is unclear how the 272 groups were selected initially, more information on the identification and selection of the self-help groups will be helpful.
2. **Response:** The 272 self help group were formed by BasicNeeds based on the concentration of people with mental health conditions. So, there was no selection. We have added a statement to clarify this on page 2 of the revised manuscript.
3. Page 3 line 95-96
   Please add the criteria used to determine which SHGs only received additional support for the second and third years.
3. **Response:** To select which SHGs received additional support in year 2 and 3, six regions were purposively selected from a total of nine, representing both Northern (Rural) and Southern (Urban) areas where programme interventions were implemented. Within each of the six regions, all districts with Self-Help Groups (SHGs) were identified, and districts were randomly selected. Additionally, SHGs within the selected district were listed on folded pieces of paper, and one SHG was randomly selected. Regions with a greater number of districts had a larger number of districts and, therefore, more SHGs included in the selection.
4. Page 5 lines 148-151
   Please add more information on what criteria was used to determine who would continue to receive support in year two and three.
4. **Response:** To select which SHGs received additional support in year 2 and 3, six regions were purposively selected from a total of nine, representing both Northern (Rural) and Southern (Urban) areas where programme interventions were implemented. Within each of the six regions, all districts with Self-Help Groups (SHGs) were identified, and districts were randomly selected. Additionally, SHGs within the selected district were listed on folded pieces of paper, and one SHG was randomly selected. Regions with a greater number of districts had a larger number of districts and, therefore, more SHGs included in the selection.
5. Page 5 lines 150-151
   How did the researcher mitigate against bias of selection of members included those that had good knowledge on the operations of the groups?

- **Response:** The study engaged the leadership for the in-depth interview as key informants. However, for the focus group discussion, selection was not based on knowledge but membership.

6. Page 6 line 210
   The author indicates that NVivo 13 was used for data analysis however the abstract indicates that NVivo 14 was used please clarify which version was used

- **Response:** NVivo 14 was used or the analysis. The inconsistency has been corrected

7. The themes in the Results section could be organised better to make it easier for the reader to follow, there seems to be information that is being highlighted about the frequency of the meetings that participants attended, stronger relationships or partnerships with other agencies including government agencies, the rights and advocacy of participants and their inclusion in meetings however this does not come out clearly when reading the section.

- **Response:**

8. Perhaps renaming the sub-headings in the section based on the most prominent theme will be helpful.

- **Response:**

9. There appears to be inconsistencies in labelling the participants to indicate which groups they belong to, it will be helpful for the authors to relook at this and label participants consistently.

- **Response:**

10. Page 10: Findings on this page seem to be referring to barriers to attending meetings and it follows on from a section that explains benefits of campaigns – organising this section better to include similar themes will allow for a more logical flow.
11. Page 10-11: Some of the quotes used have also been used on page 9, it might be helpful to avoid repeating the quotes that have already been used, organising the themes better could help avoid this.

- **Response:** This has been deleted

12. Pages 10-11: Sections on sustainability of SHG activities and challenges in sustaining activities seem to duplicate some concepts

- **Response:** In response to comments from another reviewer, this section has been deleted and the table moved to the end of the results section as summary of the study findings.

13. Page 12 Table 2 could be moved towards the end of the article as a summary of the challenges and propose solutions for all the themes that have been highlighted

- **Response:** In response to comments from another reviewer, this section has been deleted and the table moved to the end of the results section as summary of the study findings.

14. The discussion could be better organised to match the findings section to improve the flow of the section.

- **Response:** This has been re-organised accordingly

15. The reader is left wondering if a qualitative evaluation is the best way to assess effectiveness and if the study has highlighted the effectiveness of SHG through its findings, or if the study has highlighted barriers and facilitators of SHG?

- **Response:** Thank you for the comments. The researcher felt that the qualitative evaluation would provide better insight to help improve the programme. Generally, the aim of evaluation is to improve the situation, as such the researcher are of the view that
